# Context-dependent function of the transcriptional regulator Rap1 in gene silencing and activation in *Saccharomyces cerevisiae*

**DOI:** 10.1073/pnas.2304343120

**Published:** 2023-09-28

**Authors:** Eliana R. Bondra, Jasper Rine

**Affiliations:** ^a^Department of Molecular and Cell Biology, University of California, Berkeley, CA 94720

**Keywords:** epigenetics, chromatin, gene silencing

## Abstract

In *Saccharomyces cerevisiae,* heterochromatin is formed through interactions between site-specific DNA-binding factors, including the transcriptional activator Repressor Activator Protein (Rap1), and Sir proteins. Despite an understanding of the establishment and maintenance of Sir-silenced chromatin, the mechanism of gene silencing by Sir proteins has remained a mystery. Utilizing high-resolution chromatin immunoprecipitation, we found that Rap1, the native activator of the bidirectional *HML*α promoter, bound its recognition sequence in silenced chromatin, and its binding was enhanced by the presence of Sir proteins. In contrast to prior results, various components of transcription machinery were not able to access *HML*α in the silenced state. These findings disproved the long-standing model of indiscriminate steric occlusion by Sir proteins and led to investigation of the role of the transcriptional activator Rap1 in Sir-silenced chromatin. Using a highly sensitive assay that monitors loss-of-silencing events, we identified a role for promoter-bound Rap1 in the maintenance of silent chromatin through interactions with the Sir complex. We also found that promoter-bound Rap1 activated *HML*α when in an expressed state, and aided in the transition from transcription initiation to elongation. Highlighting the importance of epigenetic context in transcription factor function, these results point toward a model in which the duality of Rap1 function was mediated by local chromatin environment rather than binding-site availability.

## Significance

The coarse partitioning of the genome into regions of active euchromatin and repressed heterochromatin is an important, and conserved, level of gene expression regulation in eukaryotes. Repressor Activator Protein (Rap1) is a transcription factor that promotes the activation of genes when recruited to promoters, and aids in the establishment of heterochromatin through interactions with silencer elements. Here, we investigate the role of Rap1 when bound to a promoter in silent chromatin and dissect the context-specific epigenetic cues that regulate the dual properties of this transcription factor. Together, our data highlight the importance of protein–protein interactions and local chromatin state on transcription factor function.

Cellular identity can be defined by the array of expressed and repressed genes in a cell. Thus, two cells with identical genomes can exhibit vastly different phenotypes. Due to the wide variety of expression patterns needed for normal development and function, eukaryotic gene expression is controlled by many different processes ranging from gene-specific combinatorial effects of transcription factors to domain-wide compaction or accessibility of chromatin. Further, modifications to chromatin promote differential regulation via both the recruitment and restriction of transcriptional activators and repressors (1). The coarse partitioning of the genome into regions of actively expressed euchromatin and repressed heterochromatin is a characteristic of eukaryotic genomes and a major point of gene expression regulation (2). The stability of cell type is controlled, in large part, by the faithful propagation of cell-type-specific patterns of gene expression over cellular divisions. Breakdown of finely tuned expression programs can lead to aberrant gene expression, disease, or cell death.

In *Saccharomyces cerevisiae*, heterochromatin is controlled by the Silent Information Regulator (Sir) proteins, which assemble at the cryptic mating-type loci, *HML* and *HMR,* and the telomeres (3–5). Study of the recruitment and spread of these proteins has been fundamental in understanding the establishment and maintenance of heterochromatin (6). The canonical view of the establishment of silencing posits that Sir proteins are recruited to nucleation sites termed silencers (7–10). The *E* and *I* silencers, negative *cis-*regulatory sequences, flank both *HML* and *HMR* and are the sites from which Sir proteins spread across these loci in a sequence-independent manner. Recent evidence from our laboratory indicates that, in addition to these silencers, the promoter of *HML (HML-p)* acts as an early nucleation site of silencing (11). Common to all three of these early-recruitment loci (*HML-E, HML-I,* and *HML-p)* is the presence of a binding site for Repressor activator protein 1 (Rap1) (8, 10, 12, 13).

Rap1 is best characterized in its role as an essential transcription factor that activates hundreds of genes across the genome including the majority of ribosomal protein genes (14–18). Much of Rap1 research has focused on the activator function of the protein. In vitro studies of Rap1 classify it as a “pioneer factor”, a term used to described a class of proteins that are unique in their ability to bind to DNA in the presence of nucleosomes, establish domains of open chromatin, and facilitate binding and recruitment of other transcription factors (18–23). In addition to the Sir proteins, Rap1 functions in establishing and maintaining silent chromatin at *HML, HMR,* and telomeres by binding to silencers and recruiting Sir proteins in combination with two other silencer-binding proteins, the Origin Recognition Complex (ORC) and the transcription factor ARS-binding factor 1 (Abf1) (24–30). How Rap1 mediates two apparently opposing functions has remained a mystery.

Despite decades of research utilizing Sir-silenced chromatin as a model for heterochromatic gene repression, the fundamental question regarding the mechanism of Sir-based silencing has remained inadequately answered. In the most broad-scale model, Sir proteins form a macromolecular complex that blocks, wholesale, protein–DNA interactions in silent chromatin, including transcription factors accessing their cognate-binding sites. This model is supported by evidence of expression state–dependent cleavage and modification of enzyme recognition sites in silent or active chromatin (31, 32). A more nuanced version of this mechanism supports specific preinitiation complex interference by Sir proteins. Here, DNA-binding activators access their binding sites in Sir-silenced chromatin, but subsequent assembly of a functional preinitiation complex is somehow hindered (33, 34). Yet, other work suggests a downstream-inhibition model whereby silencing acts by prohibiting formation of mature transcripts rather than transcriptional initiation and is based on results indicating no difference in recruitment of TATA-Binding Protein (TBP) nor RNA Pol II to silent chromatin, but instead a marked absence of elongation factors and mRNA capping machinery (35, 36). Thus, the extent to which transcription machinery is occluded, and the specificity of such blockage, has remained inconclusive.

Sequence identity between the mating-type locus *MAT* and the auxiliary *HML* allows a unique opportunity to study the role of Rap1 in both silent and expressed contexts, and how local chromatin state affects function. The promoters of *MATα* and *HMLα* are identical in sequence and, thus, each contains a Rap1-binding site. Rap1 binding at *MAT* is responsible for activation of *α1* and *α2* (37, 38). The presence of this same promoter-binding site at *HML*, which is constitutively silenced, offers an opportunity to test predictions of the various models of silencing. While it is generally understood that Rap1 binding at the silencers *HML-E* and *HML-I* recruits Sir proteins to mediate silencing, the role for Rap1 at the promoter is posited to be an activator (8, 37, 38). To date, it is unclear to what extent Rap1 binds its recognition site in a heterochromatinized context, and whether it contributes to either silencing or activation of the *HML* locus.

Prior studies have been unable to query the endogenous activator at the *HML* due to the difficulty of distinguishing binding at this locus from binding at *MAT*. To better understand the dichotomy of Rap1 function, we utilized endogenous tagging of the protein in combination with high-resolution ChIP-seq and RNA measurements to characterize the contributions of Rap1 to silencing and expression at *HML*. We investigated the in vivo residence times of Rap1 to further characterize the interaction between Rap1 and chromatin.

## Results

### Rap1 Bound to the Promoter of *HML* in a Silenced State but Failed to Recruit Transcription Machinery.

**Fig. 1.**
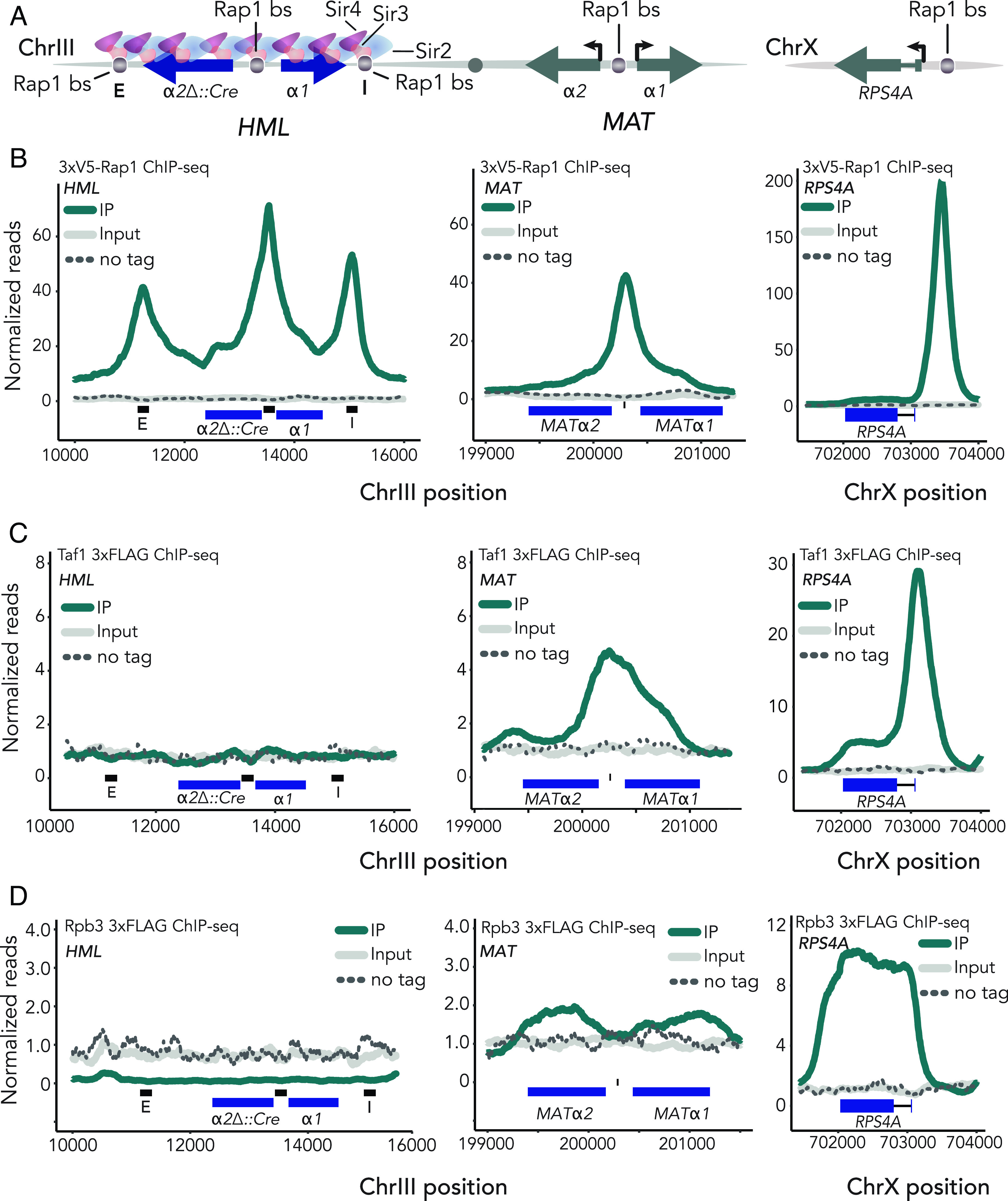
Rap1 bound the promoter of *HML* in a silenced state but failed to recruit the preinitiation complex. For all ChIP-seq experiments, read counts were normalized to the nonheterochromatic genome-wide median. IP, input, and untagged control values are plotted on the same scale. Data shown are the average of two ChIP-seq experiments unless otherwise noted. (*A*) Schematic of *HMLα* and MAT*α* on chromosome III. Rap1-binding sites at *HML-EHML-I,* and the promoter of both *HML* and *MAT* are noted. (*B*, *Left*) averaged normalized reads for ChIP-seq in two 3xV5-Rap1 samples at *HML* in *SIR* cells. Black bars represent 200 bp surrounding Rap1-binding sites at *HML-E, HML-p,* and *HML-I,* respectively. (*Middle*) same as left but showing *MAT.* (*Right*) same as (*Left*, *Middle*) but at *RPS4A.* (*C*). Same as *B* but for Taf1-3xFLAG-KanMX. (*D*) Same as *B* and *C* but for Rpb3-3xFLAG-KanMX.

Previous studies addressing the mechanism of silencing have yielded contradictory and sometimes paradoxical results, due in part to the low-resolution techniques used at the time (33–36, 39). In studies attempting to characterize the limiting steps of Pol II recruitment to a silenced locus, regions of sequence identity between *MAT, HML,* and *HMR* have interfered with the unambiguous assignment of recruitment of different factors to the loci (Fig. 1*A*). To ensure unambiguous interpretation of our results regarding recruitment to *HML*, we designed and performed experiments in strains lacking *MATα* (*mat∆)* and wherein the *α2* coding sequence at *HML* was replaced by the coding sequence for the *Cre* recombinase (*hmlα*2*∆::Cre*), thus avoiding confounding sequence identity. Similarly, to characterize enrichment at *MATα*, we performed experiments in strains lacking both *HML and HMR (hml∆ hmr∆).* In both cases, only one copy of the *α1/α2* genes was present.

We tagged endogenous Rap1 with 3xV5 at the N terminus in order to retain both its essential activating and repression functions, permitting accurate representation of Rap1 enrichment at *HML* and *MAT* (*SI Appendix*, Fig. S1 *A* and *B*). Utilizing Chromatin Immunoprecipitation followed by next-generation sequencing (ChIP-seq), we determined that Rap1 was in fact bound to the promoter of *HML* in silenced chromatin (Fig. 1*B*). Compared to the extent of enrichment at *MATp*, Rap1 was unexpectedly enriched at the silenced locus relative to the unsilenced (Fig. 1*B*).

Strong enrichment of Rap1 at the *HML* promoter under wild-type conditions was incompatible with the generalized steric-hindrance model and led us to reconsider the remaining hypotheses; either that silencing occurs at some point after the recruitment of trans-activators but before that of RNA Pol II, or silencing blocks elongation, analogous to paused RNA polymerase II in other eukaryotes (33–36, 39). To distinguish between these mechanisms, we endogenously tagged a set of proteins intimately involved in RNA Pol II-dependent transcription: TATA-binding protein-associated factor 1 (Taf1), RNA polymerase subunit B 3 (Rpb3), and elongation factor 1 (Elf1). As with our tagged Rap1, epitope tags did not affect fitness of cells with the tagged versions as the only forms of these proteins in the cell (*SI Appendix*, Fig. S1 *A* and *B*).

TFIID is one of the first factors recruited to transcription initiation sites (40–42). A subunit of TFIID, Taf1, has proposed interactions with Rap1 making it a compelling protein of interest for assessing recruitment of transcription machinery to silent chromatin (43, 44). In contrast to previous reports, TFIID showed no enrichment at *HML* in silenced chromatin (Fig. 1*C*). It was therefore unsurprising that neither a major subunit of RNA Pol II, Rpb3, nor the elongation factor Elf1, exhibited any binding to silenced chromatin (Fig. 1*D* and *SI Appendix*, Fig. S1*C*). As an internal positive control, we mapped enrichment of each protein at *MATα*, where the recruitment of each followed expected patterns; the initiation factor Taf1 was localized over the promoter, while the RNA Pol II subunit Rpb3 was enriched over the gene bodies (Fig. 1 *C* and *D*). Furthermore, all three proteins were substantially enriched at *RPS4A,* a ribosomal protein gene that is also a known Rap1 target, and were appropriately enriched over the promoters or gene bodies of open reading frames in genome-wide analyses (Fig. 1 and *SI Appendix*, Figs. S1*C* and S2). These data revealed that Sir-silenced chromatin was not entirely refractory to protein binding, but specifically to RNA pol II transcription machinery. In sum, we found robust recruitment of the endogenous activator to native Sir-silenced *HML* and narrowed the step at which silencing occurs to a point between recruitment of the activator, Rap1, and the formation of the preinitiation complex.

### Rap1 Contributed to the Maintenance of Silent Chromatin at the Native *HML* Promoter.

**Fig. 2.**
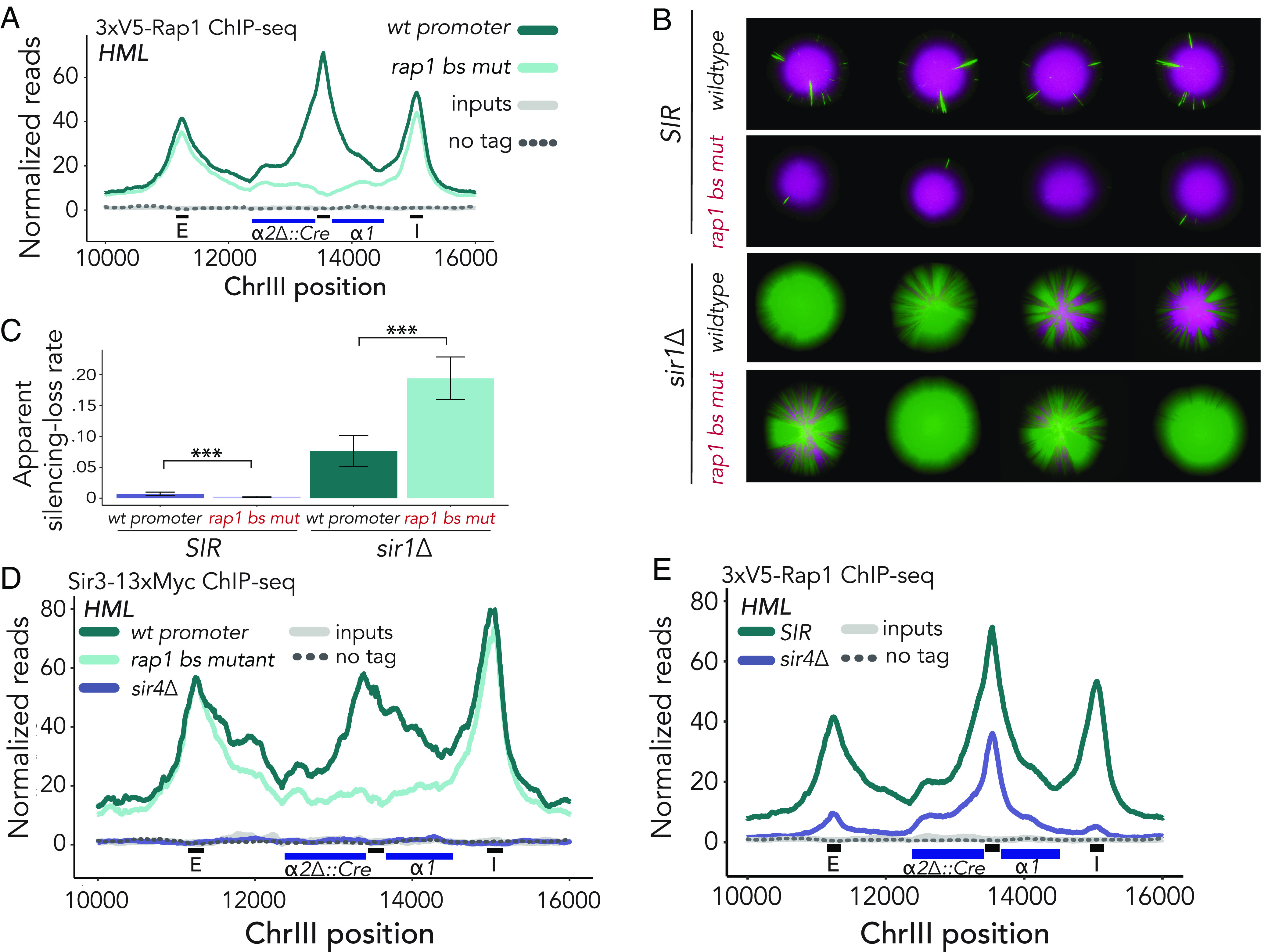
Rap1 contributes to the maintenance of silent chromatin at the native *HML* promoter. Unless otherwise stated, ChIP-seq data represented averaged reads of two biological replicates over the locus, normalized as in Fig. 1. Black bars along X-axis represent 200 bp surrounding Rap1-binding sites at *HML-E, HML-p,* and *HML-I,* respectively. IP, input, and untagged control values are plotted on the same scale. (*A*) Normalized reads mapped to *HML* in two 3xV5-Rap1 ChIP-seq experiments for wild-type and mutant Rap1-binding motif at the promoter. (*B*) Representative CRASH colonies for *SIR* and *sir1Δ* cells with wild-type and mutant Rap1-binding site at *HML-p*. (*C*) Apparent silencing-loss rate for genotypes described in *B* ± SD. The following number of events was recorded for each sample: *SIR* wt promoter (n = 271,933); *SIR* rap1 bs mutant (n = 773,105); *sir1Δ* wt promoter (n = 151,846); *sir1Δ* rap1 bs mutant (n = 90,211). *P*-values (*P* < 2.2e-16) for both comparisons were calculated using a two-sided *t*-test. (*D*) Normalized ChIP-seq reads for Sir3-13xMyc mapped to *HML* for wild-type, mutant Rap1-binding motif at the promoter, and in *sir4Δ* cells. (*E*) Normalized ChIP-seq reads for 3xV5-Rap1 mapped to *HML* in *sir4Δ* and *SIR* cells.

Given that Rap1 was enriched at the *HML* promoter in silenced chromatin, but TFIID was not, we investigated the possibility that promoter-bound Rap1 contributed to silencing the locus. We generated a strain with a two base-pair mutation in the Rap1-binding site at the promoter known to strongly decrease expression of *α1* αnd *α2* (37, 38). This mutation resulted in significant reduction of Rap1 at its consensus binding sequence within the *HML* promoter (Fig. 2*A* and *SI Appendix*, Fig. S3*D*). Introduction of this binding site mutation did not affect enrichment of Rap1 at other loci genome-wide (*SI Appendix*, Fig. S3*A*).

To evaluate the impact of the Rap1-binding site mutation (rap1 bs mutant) on silencing at *HML*, we introduced the two base-pair mutation into a previously developed strain that monitors loss-of-silencing events (45). This assay, Cre-Reported Altered States of Heterochromatin (CRASH), allows for highly sensitive measurements of loss of silencing events by expression of *HMLα2∆::Cre* and a subsequent recombination event that results in a unidirectional switch from red fluorescence to green fluorescence (Fig. 2 *B*, *Top* and *SI Appendix*, Fig. S3*B*) (45). Silencing is a robust process that fails approximately once in every 1,000 cell divisions (45, 46). To increase the level of expression to a measurable amount and broaden the dynamic range, we deleted the *SIR1* gene (*sir1∆*) in a strain with the rap1 bs mutation and the CRASH background. *sir1∆* cells exist in a bimodal expression state at *HML* (47). Recent evidence has shown that even silenced *sir1∆* cells exhibit reduced binding of all other Sir proteins across the locus (48), thereby representing a weakened heterochromatic domain. Interestingly, mutation of the Rap1-binding site at the *HML-*promoter in *sir1∆* cells did not show reduced sectoring (Fig. 2 *B*, *Bottom*). We utilized flow cytometry to quantify changes to the silenced domain observed with the CRASH assay (49). Surprisingly, in *sir1∆* cells the apparent silencing-loss rate was higher in rap1 bs mutant cells than in those with the wild-type promoter (Fig. 2*C*). Additionally, mRNA abundance measurements by reverse-transcription (RT-qPCR) of *HMLα2∆::Cre* confirmed increased *Cre* expression in *sir1∆* bs mutant cells as compared to *sir1∆* alone (*SI Appendix*, Fig. S3*C*), indicating that promoter-bound Rap1 actively contributed to silencing at *HML*. Together, these contrasting results showed that Rap1 could perform opposing functions dependent on nuances in the local chromatin environment.

**Fig. 3.**
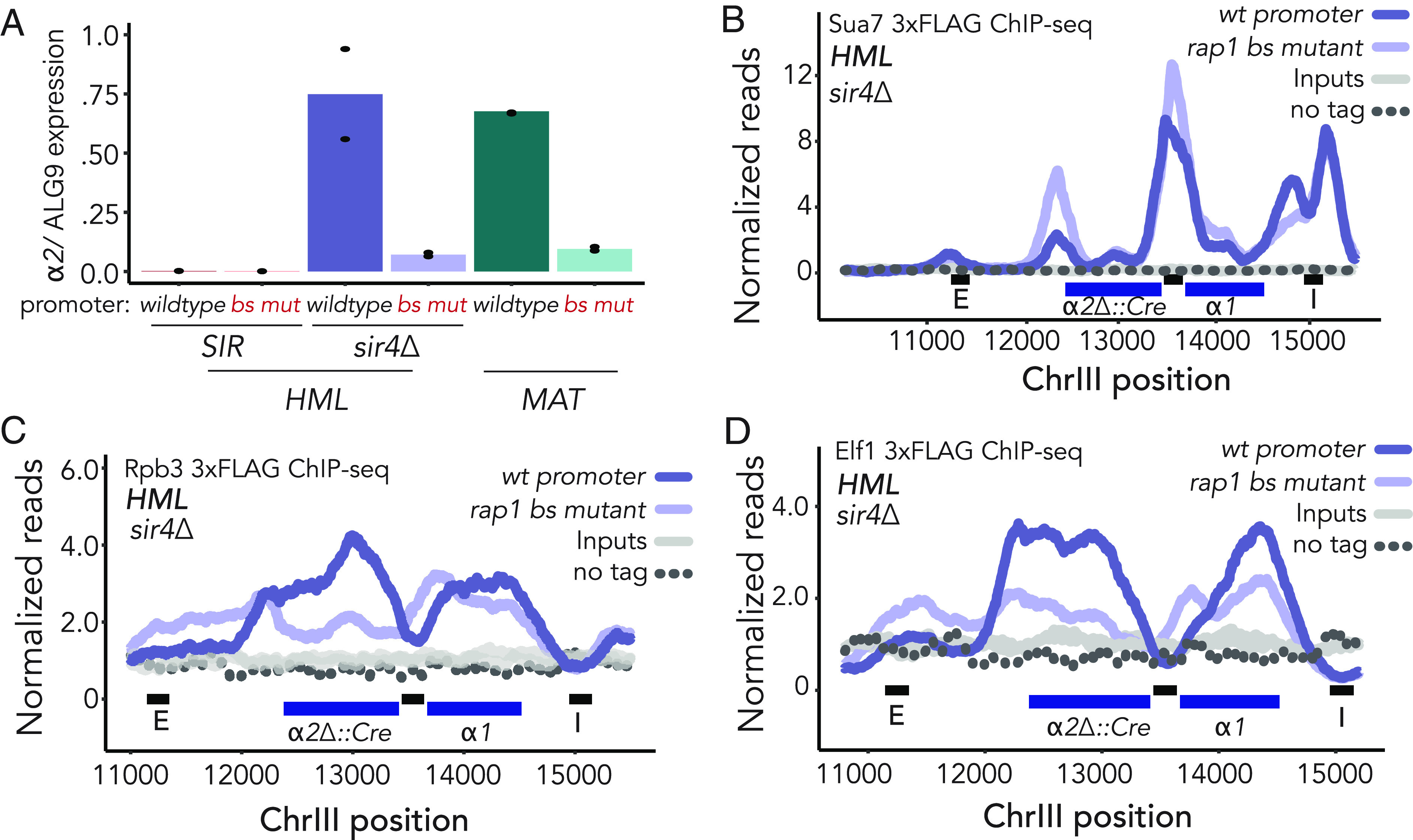
Promoter-bound Rap1 was able to activate transcription of unsilenced *α2* and aided the transition from initiation to elongation at unsilenced *HML*. For all ChIP-seq experiments, read counts were normalized to the nonheterochromatic genome-wide median. IP, input, and untagged control values are plotted on the same scale. Data shown are the average of two ChIP-seq experiments, unless otherwise noted. (*A*) RT-qPCR quantification of *α2* expression at *HML* and *MAT* normalized to control locus *ALG9.* Each plot consists of an average of two biological replicates, each represented with individual dots. (*B*) Normalized reads for ChIP-seq of Sua7-3xFLAG at *HML* in *sir4∆* cells. (*C*) Same as *B* but for Rpb3-3xFLAG-KanMX. (*D*) Same as *B* and *C* but for Elf1-3xFLAG-KanMX.

We hypothesized that promoter-bound Rap1’s intrinsic enhancement of silencing may act through stabilizing the silent domain. Therefore, we performed ChIP-seq of a Myc-tagged allele of Sir3 as a proxy for enrichment of the Sir complex across the locus. Congruous with our finding that apparent silencing-loss rate was higher in the weakened heterochromatic state of *sir1∆* cells harboring the rap1 bs mutation, Sir3 occupancy was reduced in rap1 bs mutant cells, though not to the same degree as in *sir4∆* cells (Fig. 2*D*). This reduction was particularly striking over the promoter, showing an approximate threefold reduction in Sir3 occupancy at this locus. In contrast, Sir3 enrichment at *HML-E* and *HML-I* was unaffected. Although Sir3 enrichment was reduced by deletion of the Rap1-binding site, measurements in Sir-competent cells by both CRASH (Fig. 2*B*) and RT-qPCR (Fig. 3*A*) revealed that these cells were able to maintain silencing. This result further supported a model in which interactions between Sir proteins and Rap1 cooperate to form and maintain silenced chromatin.

Cooperativity between Sir proteins and Rap1 would predict that enrichment of Rap1 at the promoter may decrease accordingly in the absence of Sir proteins. We therefore performed Rap1 ChIP-seq in *sir4∆* cells, in which the Sir complex is absent from the *HM* loci (Fig. 2*E*). Rap1 enrichment at *HML-p* in silenced cells was found to be significantly greater than that in unsilenced cells (Fig. 2*E* and *SI Appendix*, Fig. S3 *D* and *H*; Student’s *t* test *P* = 0.024). We also found a substantial decrease in Rap1 occupancy at the silencers *HML-E, HML-I,* and *HMR-E,* which each have a Rap1-binding site important for silencing, despite sequence-specific recruitment of Rap1 to these loci preceding Sir protein recruitment in canonical models for the establishment of silencing (Fig. 2*E* and *SI Appendix*, Fig. S3 *E* and *G*). To test whether diminished occupancy of Rap1 at *HML* in *sir4∆* cells was due to a disruption of the interaction between Sir4 and the C-terminal domain of Rap1, we performed ChIP-seq in *sir3∆* cells and found the results to be nearly identical (*SI Appendix*, Fig. S3 *E* and *F*). In support of this cooperativity, Rap1 enrichment at *MATα* was similar to that at unsilenced *HML* and less than the enrichment at *HML* in silenced chromatin (Figs. 1*B* and 2*E* and *SI Appendix*, Fig. S3*H*). As expected, Rap1 binding at *MATα* did not vary based on the availability of the Sir complex (*SI Appendix*, Fig. S3*G*). Collectively, these data implied that cooperative interactions existed between Sir proteins and Rap1 (Figs. 1*B* and 2*E*). Taken together, these findings established a specific contribution of promoter-bound Rap1 to silencing, where it was previously thought to have potential only for activation.

### Promoter-Bound Rap1 Activated Transcription of Unsilenced *α2* and Aided the Transition from Initiation to Elongation at Unsilenced *HML*.

Rap1 is required for transcription of *α2* and *α1* at *MAT* (38). Therefore, by abrogating Rap1 enrichment at the cognate *HML* promoter site, we presumably disrupted expression of the locus to some degree. To assess the extent to which Rap1 at the *HML* promoter had the potential to also serve as a transcriptional activator, we performed RT-qPCR of *α2* to measure mRNA expression from the *HMLα* locus in Sir+ and Sir- cells. Expression of *HMLα* was nearly undetectable in *SIR+* cells. However, in *sir4*∆ cells, which have no Sir protein recruitment to the locus, expression of *α2* from *HML* was comparable to its expression from *MAT* (Fig. 3*A*). In contrast, we saw an approximate fivefold reduction in *HMLα2* expression in rap1 bs mutant cells as compared to wild-type *HML-p,* which was comparable to the reduction caused by the same binding site mutation at *MAT* (Fig. 3*A*). This result was consistent with our finding that rap1 bs mutant cells have lower rates of sectoring than their wild-type promoter counterparts in the CRASH assay (Fig. 2*B*, second panel). These data confirmed previous work on the role of Rap1 at *MAT* (37, 38), and extended those conclusions by establishing that Rap1 contributed significantly and equivalently to expression of *α1* and *α2* at both the unsilenced *HML* and native *MAT* loci (Fig. 3*A*).

To understand which step of transcription Rap1 contributed to the most, we performed ChIP-seq of tagged transcription-associated proteins in *sir4∆* cells with and without the rap1 bs mutation at the promoter. As predicted by the decrease in gene expression (Fig. 3*A*), enrichment of major Pol II subunit Rpb3 and elongation factor Elf1 over the gene bodies of *HMLα2* and *α1* was decreased (Fig. 3 *C* and *D*). We noted, however, that rather than exhibiting the same decreased occupancy as the coding sequence, Elf1 and Rpb3 were enriched at the promoter in rap1 bs mutant cells relative to their wild-type counterparts (Fig. 3 *C* and *D*). This pattern is indicative of a failure in promoter escape, or the transition to productive elongation (50). Furthermore, we found enrichment of TFIIB subunit Suppressor of AUG 7 (Sua7) over the promoter in rap1 bs mutant cells relative to wild-type cells (Fig. 3*B*). TFIIB typically dissociates from the promoter at the initiation stage and does not travel with RNA Pol II as it transcribes (51). However, when we recapitulated the mutation in a Rap1-binding site upstream of *SST2,* another Rap1-activated gene, recruitment of Sua7 and Elf1 were markedly diminished across the entire locus (*SI Appendix*, Fig. S4 *B*–*D*). Together these findings suggested a role for Rap1 in promoter escape at actively transcribed *HML,* though this finding may be unique to the promoter architecture of the locus.

### In Vivo Rap1 Residence Time Did Not Correlate with Differences in Function at *HML* and *MAT*.

**Fig. 4.**
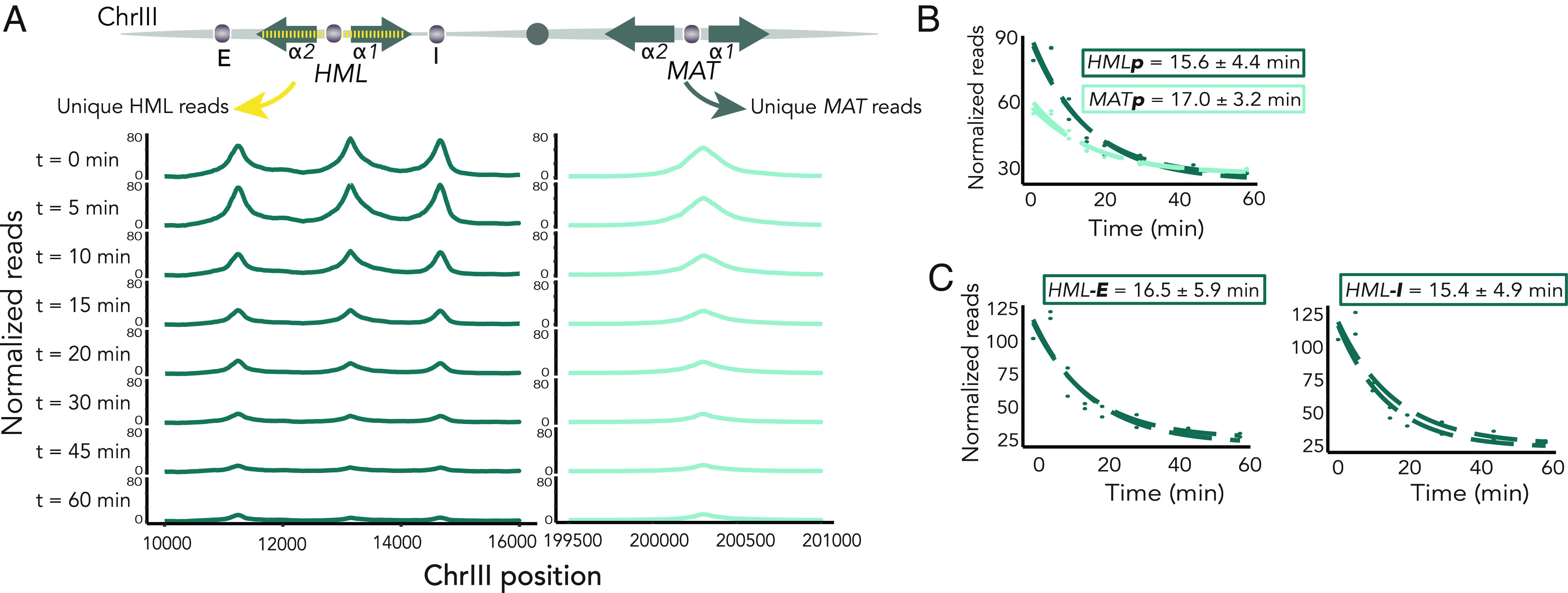
In vivo Rap1 residence time did not reflect differences in chromatin state. Decay of Rap1 occupancy at *HML* and *MAT* by Anchor Away. (*A*, *Top*), schematic of introduction of SNPs to enable unique mapping of *HML* and *MAT* in a strain that contains both. (*Below*) Rap1 enrichment by two, averaged ChIP-seq experiments at *HML* (*Left*) and *MAT* (*Right*) over time-course, plotted on the same Y-axes. (*B*) Fitted nonlinear regressions for residence times of *HML-p* and *MAT-p.* Each replicate is shown separately ± SE of average residence time. (*C*) Fitted nonlinear regressions for residence times of *HML-E* and *HML-I* as in *B*.

Considering recent focus on protein dynamics as a critical lens through which to study transcription, we hypothesized that the dynamics of Rap1–DNA interactions may vary between heterochromatin and euchromatin, due to the distinct compositions of the two structures. To test this hypothesis, we utilized the rapid nuclear depletion strategy afforded by the anchor-away technique to remove unbound Rap1 from the nucleus (*SI Appendix*, Fig. S5*A*) (52). Whereas the experiments performed earlier in this manuscript utilized strains containing either *HML* or *MAT,* we wished to directly assay apparent dwell-time at *HMLα* and *MATα* in the same cell. Thus, we generated a series of synonymous single nucleotide polymorphisms in *HML* every approximately 30 bases, spanning from E to I silencers, to allow unambiguous assignment of even short high-throughput sequencing reads to either *MAT* or *HML* (Fig. 4*A* and Dataset S2) (53). Due to the nature of the anchor-away experiment, wherein Rap1 was depleted over time, it was important to include a spike-in control for downstream analysis of the ChIP-seq data. We used cells from the closely related species *Saccharomyces paradoxus* which allowed unique mapping of sequences from each species (54, 55). Normalizing to number of reads in each sample assigned to *S. paradoxus,* we fit ChIP-seq enrichment data to a nonlinear regression model as described by the DIVORSEQ method (56) and calculated the apparent k_off_ for each peak, and thus a proxy for the in vivo residence time (*SI Appendix*, Fig. S5*A*). We characterized the fits and apparent residence times for 377 Rap1-bound loci across the genome, in replicate, at which Rap1 binding decayed over time (*SI Appendix*, Fig. S5 *B*–*G*). The residence time of Rap1 at the promoter in silent (*HML*) and active (*MAT*) chromatin was similar, although initial Rap1 enrichment was decreased at *MAT* as seen previously (Fig. 4*B*). The dwell-time of Rap1 bound to silencers was also similar (Fig. 4 *C* and *D*). These results indicated that the dual functions of Rap1 could not be attributed to differences in dynamics, but rather resulted from local chromatin contexts and possibly other protein–protein interactions.

### Genome-Wide Analysis of In Vivo Rap1 Apparent Residence Times.

**Fig. 5.**
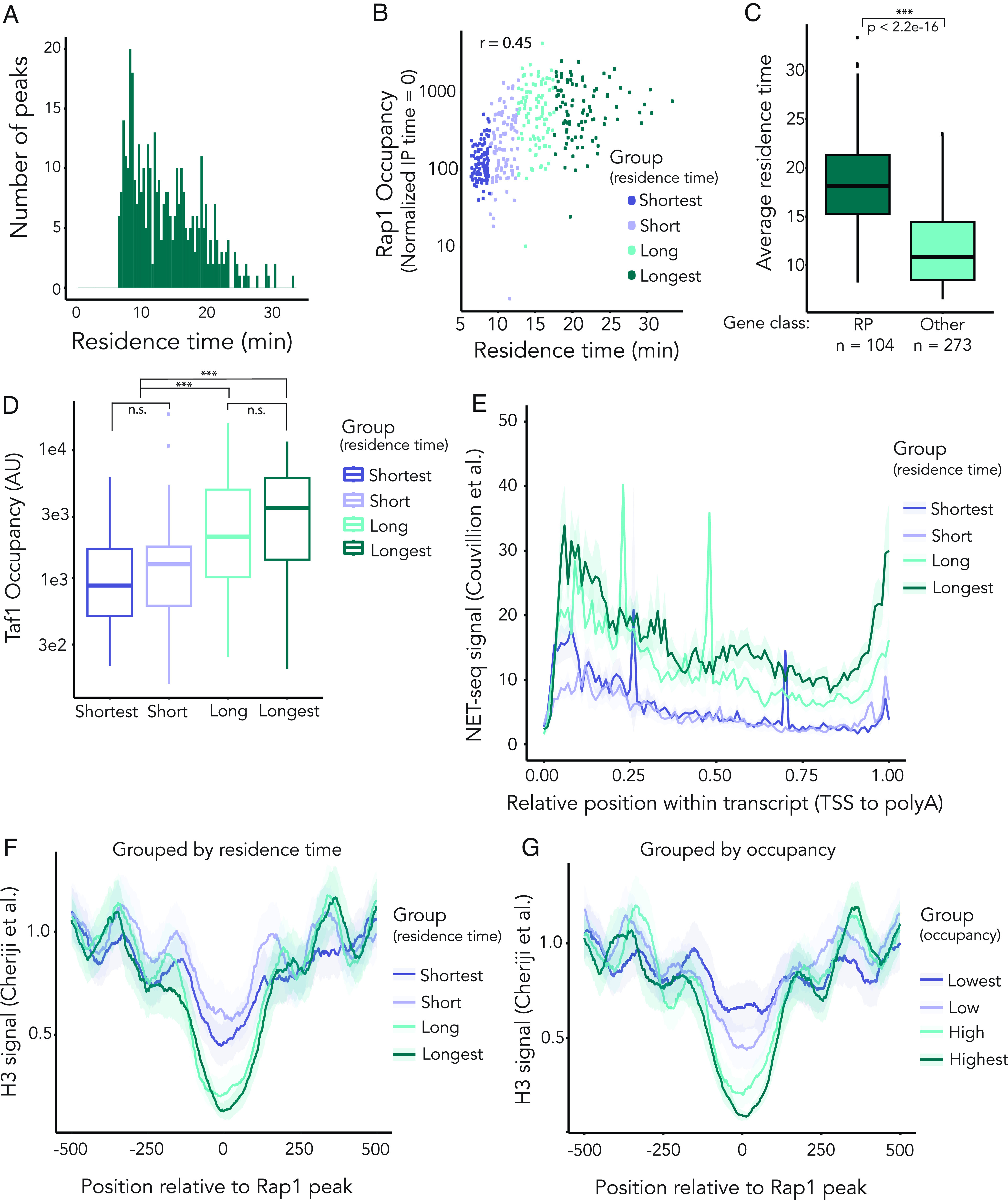
Genome-wide analysis of in vivo Rap1 apparent residence times supports and extends previous models that Rap1 dwell-time is correlated with transcriptional output. All figures comprise data obtained from the average of two biological replicates. Peak set (n = 377) was divided into quartiles based on residence time for analysis unless otherwise noted. For gene-level analyses, Rap1 peaks were assigned to ORFs for which a Rap1 peak summit was within 300 bp upstream of ORF start. (*A*) Average apparent Rap1 residence time in minutes of the 377 binding sites evaluated genome-wide. (*B*) Correlation between average Rap1 occupancy before depletion (enrichment at t = 0) and the average apparent residence times for all 377 Rap1-bound peaks (Pearson correlation *r* = 0.45, *P*-value < 2.2e-16). (*C*) Differences in apparent residence times between sites that are classified as regulating Ribosomal Protein genes (n = 104, dark green) and all other sites (n = 273, light green). The *P*-value was calculated using a Mann–Whitney U test (*P* < 2.2e-16). (*D*) Quantification, by mean apparent residence time quartile, of normalized Taf1 occupancy levels at the Rap1-binding sites. These levels are defined as the amount of Taf1 enrichment (in reads) covering the Rap1-bound loci. Significance was calculated using a one-way ANOVA followed by Tukey’s HSD test (*P* < 2.2e-16). (*E*) Mean profiles display NET-seq coverage (57) with 95% confidence intervals (CI, displayed as transparent filling) within neighboring transcript(s). Coverage was scaled according to transcript length (58). (*F*) Summary distribution plots of average H3 enrichment (59) centered on Rap1 peaks and spanning 500 bp+/−. Coverage was grouped by apparent-residence time quartiles. The CI are indicated as in Fig. 5*E*, with a transparent fill denoting 95% CI. (*G*) Same as *F*, but peaks grouped by ranked Rap1 occupancy at t = 0.

As function in silencing or activation at *HML* did not appear to contribute to, or result from, differences in Rap1 apparent off-rate, we investigated which other context-specific cues contributed to this metric. Using cutoffs similar to those previously described (56), we refined a set of 1,118 Rap1-bound regions genome-wide and ultimately measured Rap1 off-rate at 377 of these sites (Fig. 5*A* and *SI Appendix*, Fig. S6). To assess the contribution of Rap1 apparent dwell-time to function in transcriptional regulation, we selected Rap1 enrichment peaks that were within 500 bp upstream of open reading frames and assigned peaks to these respective genes. This dataset was then subdivided into quartiles based on residence time: shortest (n = 95), short (n = 95), long (n = 93), longest (n = 94).

As ChIP-seq peak heights reflect a static view of occupancy of chromatin-binding proteins, we tested the correlation between Rap1 occupancy (enrichment at time = 0) and apparent dwell-time (Fig. 5*B*). There was a significant positive correlation between these two factors (Pearson correlation coefficient *r* = 0.45, *P*-value < 2.2e-16), but much of the variation remained unaccounted for. Rap1 targets vary greatly in expression. Indeed, ribosomal protein genes whose transcripts make up nearly 60% of total mRNAs in the yeast cell (60) were enriched for longer apparent dwell-times as reported previously (Fig. 5*C*) (61). Presumably, stable Rap1 binding would allow for efficient recruitment of preinitiation complex machinery via TFIID–Rap1 interactions (44). Utilizing our previously generated dataset of Taf1 ChIP-seq as a proxy for TFIID occupancy, we compared dwell-time to Taf1 enrichment. Overall, there was a positive correlation between apparent residence time and Taf1 ChIP signal (*SI Appendix*, Fig. S6*D*, Spearman correlation coefficient ρ = 0.43, *P*-value < 2.2e-16), and a significant difference in Taf1 occupancy between the shorter and longer Rap1 dwell times (Fig. 5*D*, ANOVA, *P*-value < 2.2e-16). To investigate further the role of Rap1 binding dynamics in transcription regulation, we generated summary distribution plots (meta-gene analyses) of nascent transcripts associated with the Rap1-bound loci genome-wide. Utilizing published datasets (57, 58), we plotted nascent, elongating Pol II occupancy reported by Native Elongating Transcript sequencing (NET-seq) for each transcript proximal to a Rap1 peak. This meta-gene analysis showed that longer apparent Rap1 dwell-time correlated with greater NET-seq signal and thus an inferred higher transcriptional output (Fig. 5*E*). These data reinforced the proposed model in which longer apparent dwell-time corresponded to higher transcriptional activity, perhaps working through stable recruitment of the preinitiation complex.

Many studies identify Rap1 as an important modulator of nucleosome-free regions through interactions with the chromatin remodeling complex RSC (18, 19, 62, 63). In vitro, introduction of nucleosomes to naked DNA is anticorrelated with stable Rap1 binding (21). We therefore hypothesized that, similarly to other transcription factors, apparent Rap1 dwell-time would be inversely correlated with nucleosome occupancy, and that Rap1-binding stability was related to its role in determining nucleosome-free regions. Using a published dataset that utilized a chemical cleavage method to precisely map single nucleosomes with high-accuracy genome-wide in yeast (59), we created a meta-gene analysis mapping histone H3 positioned 500 bp upstream and downstream of each Rap1 peak (Fig. 5*F*). The averaged nucleosome occupancy for each quartile group of residence times revealed that longer dwell time corresponded to a broader nucleosome-free region, with a notably weakened -1 positioned nucleosome (Fig. 5*F*). We then compared how nucleosome occupancy corresponded to Rap1 occupancy (IP enrichment at time = 0). When positioning data were grouped by initial enrichment instead of dwell-time, we found a consistent anticorrelation between histone occupancy centered over the Rap1 peak and relative Rap1 occupancy (Fig. 5*G*). As total enrichment is a function of both on-rate and off-rate, we surmised that the differences we observed in correlations between nucleosome occupancy and Rap1 enrichment versus apparent dwell-time may have reflected differences in on-rate. In sum, these data supported and extended the hypothesis that Rap1 binding and nucleosome occupancy were inversely correlated in vivo, and connected this attribute to transcriptional output.

## Discussion

This study explored the enigmatic ability of Rap1 protein to function in both gene activation and repression. Earlier work discounted the possibility that the function of Rap1 was determined by subtle differences in its recognition sequence (64). Other work suggested that Sir-silenced chromatin could inhibit binding of at least some proteins to their recognition sites (33, 35, 36, 39). In contrast, our findings established that silenced chromatin did not block Rap1’s recognition of its binding site in silent chromatin. Instead, our data revealed a more nuanced and unexpected way in which Rap1 and Sir proteins mutually reinforced each other in the assembly of silenced chromatin.

Rap1, bound to its recognition sequence within the bidirectional promoter between *HMLα1* and *HMLα2,* served opposing functions depending on the state of the surrounding chromatin. The *HML* promoter-binding site was robustly enriched for Rap1 in the presence of Sir proteins. In contrast, transcription initiation factors were undetectable (Fig. 1). Strikingly, Rap1 presence at the bidirectional *HML* promoter resulted in increased enrichment of Sir proteins at *HML*, and vice versa. Moreover, *HML-*promoter-bound Rap1 contributed to the stability of silent chromatin when weakened, as is the case in the *sir1∆* genetic background (Fig. 2). In addition, we determined a possible role for Rap1 in promoting the transition from initiation to elongation at the unsilenced *HML*, which may be dependent on its role in nucleosome positioning and the unique architecture of this bidirectional promoter.

Our results agreed with earlier work that Sir-silenced chromatin can inhibit some proteins from binding their recognition sequences (33, 34, 36, 39). Despite ample Rap1 enrichment at a Sir-silenced *HML,* we found no evidence of the preinitiation complex bound at the *HML* promoter in silent chromatin, nor any indication that silencing acts by blocking Pol II elongation (Fig. 1). Our study used tagged forms of Taf1, Sua7, Rpb3, and Elf1, each expressed from their native promoter as proxies for TFIID, TFIIB, RNA Pol II, and elongation machinery, respectively. Previous reports find TBP and TFIIH to be present in the context of Sir-silenced chromatin (36). The earlier study utilized ChIP in conjunction with PCR and did not provide the resolution of the current study. In summary, our results demonstrated that Sir-silencing occurred through a preinitiation complex interference mechanism, whereby the presence of Sir proteins competitively inhibited the ability of Rap1 to recruit the transcription machinery.

While apparent off-rates for Rap1 did not differ between silenced and unsilenced chromatin, we found greater enrichment of Rap1 in the presence of Sir proteins both at the promoter and silencers of *HML* (Figs. 1, 2, and 4). The experimental setup of our studies did not allow for direct comparison to dwell-times at *HMR*. However, we found further evidence of a positive feedback mechanism between binding of Sir proteins and Rap1 at the *HMR-E* silencer, where Rap1 enrichment was diminished in the absence of Sir proteins (*SI Appendix*, Fig. S3*G*). Taken together, we inferred that the enhanced binding of Rap1 in the presence of Sir proteins was consistent with an increase in the frequency of Rap1 binding, or on-rate, in this context. However, inferences regarding off-rate through the anchor-away method are limited by the method affecting primarily the loss of free Rap1, and any changes in on-rate are speculative. Nevertheless, our dwell-time data were consistent with those described by a related technique that reports on local competition between Rap1 molecules (61). Our data supported a hypothesis that interactions between Sir proteins and Rap1 resulted in greater recruitment of both to silenced chromatin than would be achieved by the affinity of Rap1 for its binding site alone.

The *sir1∆* genotype has long served as a case study in epigenetics (47, 65). Surprisingly, we found the apparent silencing-loss rate in *sir1∆-rap1* bs mutant cells to be greater than in a *sir1∆* alone, while the opposite was true in *SIR* cells (Fig. 2 *B* and *C*). In the *sir1∆* mutant, the density of Sir proteins at the silent locus is less than in wild type, creating a paucity of Rap1-Sir interactions and, more broadly, a decrease in stability of the silent locus (48). Combined, these data reflected the importance in Rap1-Sir protein interactions, particularly over the promoter, in the weakened *sir1∆* silent domain. The weakened interactions may allow for more opportunities for transcription machinery to interact with Rap1 and shift the balance in favor of transient derepression, as is supported by our finding that loss-of-silencing rate increased in *sir1∆* strains lacking promoter-bound Rap1 (Fig. 2 *B* and *C*).

By indirect means, the activation domain of Rap1 has been shown to interact with various TFIID components (44, 66). Despite the activation domain and C-terminal interaction domain appearing to be nonoverlapping by those analyses, one explanation for the occlusion of the preinitiation complex from Sir-silenced chromatin would be if binding of Sir proteins to Rap1 rendered the activation domain inaccessible to TFIID. Our data were compatible with the idea that interactions between Rap1 and Sir proteins are mutually exclusive to interactions between Rap1 and TFIID subunits. In this model, the balance of silencing versus activation would tip toward fully silenced as the local concentration of Sir proteins increased. This proposed mechanism is reminiscent of the model for repression by the Cyc8-Tup1 complex, which appears to mask the activation domains of several activator proteins (67). However, there are contradictory findings regarding the specificity of the Sir-Rap1 interaction for repression. Replacing the bidirectional promoter with a Gal-inducible *GAL1* promoter fails to maintain silencing at *HML* in the presence of galactose, whereas replacement with an exogenous T7 bacterial promoter maintains silencing, and Sir proteins continue to act as a significant barrier to transcription (34). Notably, in *K. lactis,* Reb1 replaces Rap1 at the *HMLα* promoter and silencers, and functionally silences the *HM* loci (68, 69).

In agreement with previous findings of other transcription factors, our data showed that nucleosome positioning was correlated with Rap1 dwell-time (56). In our anchor-away dataset, Rap1-bound loci with the longest apparent residence time were characterized by a well-defined nucleosome-free region centered on the Rap1-binding site, and a broader nucleosome-depleted region with an unstable -1 positioned nucleosome (Fig. 5*F*). Conversely, loci with shorter dwell-times corresponded with a narrowing of the nucleosome-free region centered on the Rap1 peak, and an associated relative enrichment in histone occupancy over these peaks (Fig. 5*F*). We identified a further anticorrelation between nucleosome occupancy and Rap1 occupancy (enrichment at t = 0, Fig. 5*G*), suggesting that Rap1 binding in the presence of a nucleosome was less stable, as is reported in vitro (21). By taking both the static occupancy measurement and the apparent dwell-time function into account, we inferred a variable on-rate that supports this hypothesis. These data indicated that nucleosome positioning relative to Rap1 peak summit played an important role in determining both the level to which Rap1 was enriched at those sites, and the apparent residence times once bound. However, it would be equally valid to infer that strong Rap1-binding depleted nucleosomes to a greater extent than weak Rap1 binding.

Rap1 occupancy is an important determinant of the size and patterning of the nucleosome-depleted regions to which it binds, while removal of Rap1 may result in remodeling of the nearby chromatin (18, 62, 63, 70–72). Alteration to the nucleosome-depleted region of the bidirectional promoter in the absence of Rap1 could generate a block to productive transcription. We observed a narrowed Taf1 peak in rap1 bs mutant cells, which may indicate reduced access of TBP to the TATA motif (*SI Appendix*, Fig. S4*A*). Rap1 binding is necessary for downstream recruitment of the chromatin remodeler complex RSC to maintain a nucleosome-depleted region (19). Without Rap1 binding, and subsequent recruitment of chromatin remodelers, these sites may be less accessible to subunits of TFIID and other preinitiation complex machinery. Furthermore, in the context of unsilenced *HML,* Elf1 and Rpb3 appeared to pile up over the bidirectional promoter in the absence of Rap1, which indicated a blockade in the progression of transcription (Fig. 3). Our finding that the switch from RNA Pol II initiation to elongation was hindered in the absence of Rap1 could reflect a change in the promoter architecture upon removal of Rap1. With the experiments performed here, we cannot ascribe kinetic determination to this finding and can only suggest that canonical progression of transcription initiation to elongation may be altered in the absence of Rap1 at *HML*. This phenomenon may be unique to the specific promoter architecture of the mating-type locus (*SI Appendix*, Fig. S4 *B*–*D*).

Based upon the positive correlation between Rap1 residence time and enrichment of Taf1, we hypothesized that the variability in expression strength at Rap1-bound loci genome wide may be reflected in part by variability in Rap1 dwell-time (Fig. 5*D* and *SI Appendix*, Fig. S5*G*). Furthermore, nascent transcript abundance correlated with Rap1 dwell-time (Fig. 5*E*). These findings confirmed and extended previous models suggesting that the role of Rap1 in transcriptional activation was dependent, in part, on Rap1 binding dynamics (19, 62, 63, 73). Apparent dwell-time measurements may also be a function, more broadly, of protein–protein interactions and binding-site affinities. These may include Sir-Rap1 interactions at the silent loci, and TFIID-Rap1 interactions elsewhere. While increased transcriptional output may correlate with longer dwell-times, it is possible that increased frequency of interactions with Sir proteins would appear similarly, thus explaining the similarity in apparent dwell times at silenced *HML* and active *MAT.* In summary, apparent residence time may be attributed to competition with nucleosomes, with the availability of a stable nucleosome-depleted region allowing for higher rates of transcription.

Rap1 has been described as a pioneer factor with the ability to access cognate binding sites in the presence of a nucleosome array in vitro (21, 74). Rap1 bound to its promoter site in Sir-silenced chromatin extends this view in vivo (Fig. 1). In a broader context, pioneer factors are typically utilized by the cell during periods of drastic genomic restructuring, such as during fertilization in metazoans (22, 75). In their haploid life cycles, yeast continuously undergo chromatin landscape restructuring in the form of DNA replication during replicative aging. Furthermore, it is important for the single-celled organism to readily adapt to environmental stresses. Rap1 is necessary for the Gcn4-mediated regulation of ribosomal protein genes, which occurs upon amino acid starvation (76). Perhaps the downstream effect of Rap1-mediated nucleosome-free regions is to more readily enable the genome to activate certain genes under stress conditions by promoting the transition from transcription initiation to elongation.

In summary, we found that Rap1 has a complex and context-dependent role in the regulation of gene expression, with the ability to both stabilize the Sir-silencing complex at a silenced promoter and promote transcriptional elongation at the same locus in the absence of Sir proteins. In this way, Rap1 can be compared to Glucocorticoid receptor, a transcription factor well studied for its context-specific roles in vertebrate gene regulation (reviewed in refs. 77 and 78), and Ume6, a meiotic regulator that can act as a repressor or activator depending on its cofactors (79). Like the glucocorticoid receptor, one possible explanation of how Rap1 may be able to bind DNA in heterochromatin but not recruit transcription machinery may be the presence of posttranslational modifications to the Rap1 protein. In a thematically similar concept, recent data reveal differential phosphorylation of Clr4^SUV39H^ correlates with a switch in methylation state of H3K9 in *S. pombe* (80, 81).

This work highlights the many modes of epigenetic regulatory mechanisms integrated by cells to give rise to a spectrum of context-specific, finely tuned gene expression patterns. Beyond the role of Rap1 in *S. cerevisiae,* these findings have implications in eukaryotic regulation of cell-type fidelity across cell divisions. Dual-function transcription factors can be recruited to promoters and, in a context-dependent manner, serve as coactivators or corepressors to finely tune gene expression, in part through the effects of local concentration of interaction partners. In conclusion, these findings provide insights into the mechanisms of gene expression and highlight the importance of considering the context in which transcription factors function.

## Materials and Methods

### Yeast strains.

Strains used in this study are listed in Dataset S1. All strains were derived from the *S. cerevisiae* W303 background (except JRy15212 which was derived from *S. paradoxus YPS138*) using standard genetic techniques and CRISPR-Cas9 technology (82–84). Deletions were generated using one-step replacement with marker cassettes (85, 86). Details of strain construction for epitope-tagged proteins and mutants can be found in *SI Appendix*, *Supplementary Methods*. Relevant oligonucleotides and the sequence for the unique *HML* used in Figs. 4 and 5 can be found in Dataset S2.

### CRASH Colony Imaging.

Colonies were plated onto 1.5% agar plates containing yeast nitrogen base without amino acids, 2% dextrose, and supplemented with complete supplement mixture (CSM)-Trp to minimize background fluorescence. Colonies were incubated for 5 to 7 d at 30 °C, then imaged as described in ref. 87.

### Flow Cytometry and Calculations of Apparent Loss-of-Silencing Rate in CRASH Strains.

This experiment was carried out as described in refs. 49 and 87. The apparent silencing-loss rate was calculated as previously described (49, 87) (*SI Appendix*, *Supplementary Methods*).

### RNA Extraction and RT-qPCR.

RNA extraction and RT-qPCR was carried out as in ref. 53 (*SI Appendix*, *Supplementary Methods*). Each reaction was performed in triplicate, with the matched nonreverse-transcribed sample run simultaneously. cDNA abundance was calculated using a standard curve and normalized to the reference gene *ALG9.* Oligonucleotides used for qPCR are listed in Dataset S2.

### Chromatin Immunoprecipitation, ChIP-qPCR, and Library preparation.

For ChIP-seq experiments (Figs. 1–3), cells were grown in YPD overnight in 5 mL cultures then back-diluted to a concentration of OD600 ~ 0.1 in 50 mL YPD the following day. Cells were grown to mid-log phase (OD600 ~ 0.6 to 1.0) and ~5 × 10^8^ cells were cross-linked in a final concentration of 2% formaldehyde at room temperature for 15 min. The formaldehyde was quenched using a final concentration of 1.5M of Tris for 5 min.

For anchor-away ChIP-seq experiments (Figs. 4 and 5), cells were grown overnight and back-diluted as above, then grown for two-three doublings and collected at OD600 ~ 0.8. Rapamycin (LC Laboratories) was added to a final concentration of 7.5 µM. Additions of rapamycin were staggered such that all time points were ready at the same OD. Samples were fixed and quenched as above. 5% S. *paradoxus* cells by OD were spiked into each *S. cerevisiae* sample and processed according to the chromatin immunoprecipitation protocol.

Cell lysis and chromatin immunoprecipitation were performed as described in ref. 53. Details can be found in *SI Appendix*, *Supplementary Methods*. For all samples, ~850 μL soluble chromatin were collected for immunoprecipitation, and 50 μL was reserved for Input. For all ChIP samples, 50 µL DynaBeads Protein G magnetic beads (ThermoFisher Scientific) per sample were equilibrated by washing 5× in FA Lysis buffer. IP for 3xV5-Rap1 was performed using 5 μL mouse monoclonal V5 (ThermoFisher Scientific). For all 3xFLAG-tagged proteins, IP was performed using 5 μL mouse monoclonal anti-FLAG® M2 antibody (Millipore Sigma). Samples were eluted in 100 µL TE + 1% SDS. Beads and elution buffer were incubated at 65 °C overnight to reverse cross-linking, followed by treatment with RNaseA and Proteinase K. DNA was purified using a QIAquick PCR purification kit.

For ChIP-qPCR, ChIP samples were diluted 10-fold, and input samples were diluted 100-fold in nuclease-free water. Reactions were set up in triplicate and run using the same reagents and parameters as for RT-qPCR above. Abundance was calculated using a standard curve for each primer set, and the ratio of IP/Input was plotted. Oligonucleotides used for this experiment can be found in *SI Appendix*, Table S2.

Libraries were prepared for high-throughput sequencing according to the manufacturer’s recommendations using the Ultra II DNA Library Prep kit from New England Biolabs (NEB). Samples were multiplexed and paired-end sequencing was performed using either a MiniSeq or NovaSeq 6,000 (Illumina).

### Alignment and Mapping.

Sequencing reads were aligned using Bowtie2, using options = “--local --soft-clipped-unmapped-tlen --no-unal --no-mixed --no-discordant” (88) to a reference genome. For standard ChIP-seq experiments (Figs. 1–3 the genome file was derived from SacCer3 and customized as described in *SI Appendix*, *Supplementary Methods*. Analysis was performed using custom scripts derived from ref. 53. For coverage calculations in *SI Appendix*, Fig. S2*A*, peak summits were defined by MACS3 callpeak, using a cutoff of q < 0.01. The positional information and coverage for these peaks can be found in Dataset S3.

For anchor-away experiments, a custom, concatenated hybrid genome was generated using modified SacCer3 (unique *HML* sequence, *hmr∆*) and the *S. paradoxus* genome CBS432 (genbank). Reads were aligned as above. *S. paradoxus* read count served as the normalization factor for each sample. Normalization data be found in Dataset S4.

All displays of ChIP-seq normalized coverage over a defined region were displayed using a custom Rscript and ggplot2 available on the associated GitHub.

### Peak-Calling and Filtering for Anchor-Away Experiments.

We followed the framework for peak calling and filtering laid out in ref. 56. MACS peak filtering was performed to identify regions of distinct peaks across the *S. cerevisiae* genome in control samples (DMSO-IP, time 0) using a no-tag control sample as the input over which the program defined peaks. Summits were defined using the callpeak function and options “-f BAMPE -g 1.2e7 -q 0.01 --keep-dup=auto -B --call-summits”, identifying 1,118 Rap1-bound regions genome-wide. Peaks were defined as 150 bp on either side of the summit. We counted read coverage over each region in duplicate Rap1-depletion samples, and these values were normalized to the *S. paradoxus* read counts per sample as described above. A table containing positional information for these peaks, and the normalized count data, can be found in Dataset S5.

Peaks at each locus were fit using the exponential decay model described in ref. 56, filtering for peaks with *p*-value log(k_off_) < 0.05. The fits were done in R with the nls function using the formula: “nls(ChIP ~SSasymp(time, yf, y0, log_koff)” (*SI Appendix*, *Supplementary Methods*). The 377 peaks used in the analyses for Fig. 5 and *SI Appendix*, Figs. S5 and S6 represent peaks that fit the nonlinear regression model and were within 300 bp upstream of an ORF and/or located in the subtelomeric region (defined as 15 kb from the ends of chromosomes). A table containing the calculated fit of the decay curves, average residence time, and further classifications can be found in Dataset S6.

### Other Datasets.

H3 occupancy genome-wide for analysis of the relationship between Rap1 apparent dwell-time or Rap1 enrichment to nucleosome position was downloaded from GEO Accession GSE97290 (59).

Transcript isoforms were defined using a TIF-seq dataset (GEO Accession GSE39128) (58). We then averaged the corresponding NET-seq signal from four biological replicates in GEO Accession GSE159603 (57).

## Supplementary Material

Appendix 01 (PDF)

Dataset S01 (XLSX)

Dataset S02 (XLSX)

Dataset S03 (XLSX)

Dataset S04 (XLSX)

Dataset S05 (XLSX)

Dataset S06 (XLSX)

## Data Availability

All ChIP-seq raw and processed data have been deposited in GEO (GEO Series GSE227763) (89); Custom code for analysis data have been deposited in GitHub (https://github.com/elianabondra/Bondra_and_Rine_2023) (90). Previously published data were used for this work [Fig. 5 *F* and *G* - H3 occupancy published in (59 and 91) (https://doi.org/10.1186/s13059-018-1398-0) - GSE97290. *SI Appendix*, Fig. S2 and Fig. 5*E* - TIF-seq published in (58 and 92) (https://doi.org/10.1038/nature12121) - GSE39128; Fig. 5 *F* and *G* - NET-seq published in (57 and 93) (https://doi.org/10.7554/eLife.78944) - GSE159603].
