# Supplementary material for: Context-dependent function of the transcriptional regulator Rap1 in gene silencing and activation in Saccharomyces cerevisiae
Source: Proc Natl Acad Sci U S A. 2023 Sep 28;120(40):e2304343120. doi: 10.1073/pnas.2304343120 (PMC10556627; doi:10.1073/pnas.2304343120)
Supplement: Supplementary file 1 — Appendix 01 (PDF) [file pnas.2304343120.sapp.pdf]

PNAS

# Supporting Information for

## Context dependent function of the transcriptional regulator Rap1 in gene silencing and activation in *Saccharomyces cerevisiae*

*Eliana R Bondra and Jasper Rine**

**Author Affiliations:**
Department of Molecular and Cell Biology, University of California, Berkeley, Berkeley, CA 94720, United States

*For correspondence: Jasper Rine
Email: jrine@berkeley.edu

**This PDF file includes:**

Supporting text – Supplementary Methods
Figures S1 to S6
Legends for Datasets S1 to S6
SI References

**Other supporting materials for this manuscript include the following:**

Datasets S1 to S6

All ChIP-seq datasets (raw and processed) are available at NCBI Gene Expression Omnibus (GEO): Series GSE227763.

Custom codes used for analysis are available at https://github.com/elianabondra/Bondra_and_Rine_2023

## Supplementary Methods

### Yeast strain construction

C-terminal tags (Rpb3-3xFLAG:KanMX, SUA7-3xFLAG-KanMX, ELF1-3xFLAG:KanMX, TAF1-3xFLAG:KanMX) were generated by amplifying the 3XFLAG::KanMX sequence from pJR2601 (p3FLAG-KanMX; (1)) with primers that included 40 bp of sequence identity with either side of the amplicon, followed by a transformation. The 3xV5-Rap1 N-terminally tagged allele was generated by amplifying the 3xV5 sequence from pJR3191 pFA61-3xV5-NatMX6 with sequence identity to the N-terminal insertion site on either side, then integrated using CRISPR-Cas9 technology as described (2). Rap1 was tagged N-terminally to avoid genetic manipulations of the Rap1 C-terminus, since doing would likely have interrupted interactions with Sir proteins or Rap1 interacting factors and thus obscured our interpretations in the context of silent chromatin. The rap1 binding site mutation was generated by using CRISPR-Cas9-mediated targeting of the *HML-p* sequence coupled with an oligonucleotide extension that incorporated the 2 bp mutation and obliterated the PAM sequence. The mutant allele of *HML* that included synonymous SNPs was used to distinguish sequencing between *MAT*α and *HMLα* in short sequencing reads, utilized in the Anchor Away experiments, was generated by cloning together synthetic DNA gene blocks (Integrated DNA Technologies) and a previously published allele of *HML* (3), and integrated using CRISPR-Cas9 technology. We recapitulated the Rap1 Anchor Away strain first published in (4) by amplifying the 2xV5-FRB sequence from a plasmid and integrating it, at a sequence corresponding to amino acid 134 in the *RAP1* CDS, into the Anchor Away parent strain (gifted from Craig Peterson, originally from (5)) by CRISPR-Cas9. The *S. paradoxus* strain was generated similarly, but transformed into YSP138 (a gift from the Brar/Ünal labs).

### Validation of epitope-tagged strains

Tagged strains were confirmed by PCR and Sanger sequencing, as well as Immuno-blot analysisTo address the possibility that endogenous tagging of the proteins studied impacted viability or silencing, growth-curves for representative strains were conducted over a 24 hour window. We found no difference in growth rates between wild type yeast and any of our endogenously-tagged strains (figure S1A). Furthermore, introduction of epitope tags to representative strains resulted in no silencing defects as measured by RT-qPCR of *HML*α2Δ*::Cre* (figure S1B).

### Growth and preparation of CRASH cells for flow cytometry

To summarize: strains were streaked for single colonies on YPD, with multiple single colonies used as technical replicates for each sample. Strains were then back-diluted in growth medium containing G418 to select for cells that had not yet lost silencing. Cells were diluted and grown in liquid CSM until mid-log phase and harvested by centrifugation, then resuspended in PBS at approximately 0.5 OD. Samples were processed as described in (6).

### Calculations of apparent loss-of-silencing rate in CRASH strains

Flow cytometry was done on a BD LSR Fortessa using the BD FACSDiva software (BD Biosciences) and FITC and PE-TexasRed filters. Events were analyzed and processed using FlowJo Software (BD Life Sciences) and the flowAI R package (7) as described in Fouet and Rine 2023. Samples were grouped by population; GFP+ RFP+, GFP+ RFP-, GFP- RFP+ and GFP- RFP-. The apparent silencing-loss rate was calculated by quantifying the number of cells that were transitioning from RFP to GFP expression (those expressing both RFP and *GFP*), divided by the sum of all cells still expressing RFP (RFP+ GFP+ and RFP+ GFP-) (2, 6, 8).

**RNA extraction and RT-qPCR**

Briefly, at least ~2x10$^7$ cells were grown and collected by centrifugation for each sample. RNA was purified using the RNeasy Mini Kit (Qiagen 74104; Hilden, Germany) according to manufacturer's instructions, including on-column DNase digestion (Cat No. 79254). RNA was quantified by NanoDrop, and 2mg of RNA was reverse transcribed using SuperScript III reverse transcriptase (Thermo Fisher Scientific catalog number 18080044) and an 'anchored' oligo-dT primer. A matched non-reverse-transcribed sample was generated simultaneously. The DyNAmo HS SYBR Green qPCR kit (Thermo Fisher Scientific F410L), including a Uracil-DNA Glycosylase (Thermo Fisher Scientific EN0362) treatment, was used for qPCR and samples were run using an Agilent Mx3000P thermocycler.

***S. paradoxus* spike-in**

A Rap1-V5 tagged strain of *S. paradoxus* was grown in parallel and fixed at the same saturation. As the *S. paradoxus* cells did not contain all other components of the Anchor-Away methodology, notably the *tor1-1* mutation, they were not exposed to rapamycin. After fixation, cells were washed twice in ice-cold TBS.

**Customization of genome for alignment**

For standard ChIP-seq experiments (figures 1-3), custom genomes were generated from SacCer3 and modified to include, where appropriate, the mutant *HML-p* rap1 binding site mutation, *hmlα2::Cre, mat*Δ*,* and *hmr*Δ*,* or *hml*Δ *hmr*Δ in the case of *MATα* strains. Sequence information for these genomes can be found on the associated GitHub.

**Cell lysis and chromatin isolation**

Cells were washed twice in ice-cold TBS and twice in ice-cold FA lysis buffer (50 mM HEPES, pH 7.5; 150 mM NaCl, 1 mM EDTA, 1% Triton, 0.1% sodium deoxycholate) + 0.1% SDS + protease inhibitors (cOmplete EDTA-free protease inhibitor cocktail, Sigma-Aldrich 11873580001). Cell pellets were then either flash frozen or lysed. For lysis, cell pellets were resuspended in 800uL FA lysis buffer + 0.1% SDS and ~500 µL 0.5 mm zirconia/Silica beads (BioSpec Products; Bartlesville, OK) were added. Cells were lysed using a FastPrep-24 5G (MP Biomedicals; Irvine, CA) with 6.0 m/s beating for 20 s followed by 2 min on ice, repeated four times total. Lysate was transferred to a new microcentrifuge tube, and beads were rinsed with 300uL FA lysis buffer + 0.1% SDS and the remaining lysate was transferred to the same microcentrifuge tube. The cell lysate was transferred to 15 mL Bioruptor Pico tubes along with ~200µL of the corresponding sonication beads (Diagenode C010200031) and sonicated using a Bioruptor Pico (Diagenode B01060010) for 10 cycles of 30 s ON followed by 30 s OFF. After sonication, samples were spun at 4°C for 30 min at 17 k RCF to pellet cellular debris, and ~900 µL of the chromatin-containing supernatant was saved.

**Chromatin Immunoprecipitation and sample washes following overnight immunoprecipitation**

Beads and antibodies were incubated for >3 hours in an end-over-end rotator at 4°C and then rinsed once with 500uL FA Lysis buffer + 0.1% SDS + 0.05% Tween. All immunoprecipitations were performed in an end-over-end rotator at 4°C overnight in the presence of 0.5 mg/mL BSA (NEB B9000S). Each sample was washed in the following manner, with ~5 minutes washing by incubating on an end-over-end mixer between each step: 2x washes with FA Lysis + 0.1% SDS + 0.05% Tween; 2x washes with Wash Buffer #1 (FA Lysis buffer + 0.25 M NaCl + 0.1% SDS + 0.05% Tween); 2x washes with Wash Buffer #2 (10 mM Tris, pH 8; 0.25 M LiCl; 0.5% NP-40; 0.5% sodium deoxycholate; 1 mM EDTA + 0.1% SDS + 0.05% Tween); and 1x wash with TE + 0.05% Tween.

After overnight incubation at 65°C, 5 uL 10mg/mL RNase A was added to each sample and incubated for 1 hr at 37°C. 10 µL of 800 U/mL Proteinase K (ThermoFisher Scientific, EO0491) was then added to each ample and incubated for 1 additional hour at 65°C.
Each sample was washed in the following manner, with ~5 minutes washing by incubating on an end-over-end mixer between each step: 2x washes with FA Lysis + 0.1% SDS + 0.05% Tween; 2x washes with Wash Buffer #1 (FA Lysis buffer + 0.25 M NaCl + 0.1% SDS + 0.05% Tween); 2x washes with Wash Buffer #2 (10 mM Tris, pH 8; 0.25 M LiCl; 0.5% NP-40; 0.5% sodium deoxycholate; 1 mM EDTA + 0.1% SDS + 0.05% Tween); and 1x wash with TE + 0.05% Tween.

**Peak calling for figure S2A**

Peaks were defined as 150bp on either side of the summit. Peaks were ranked by fold-enrichment and we counted read coverage over the 500 most enriched regions using featureCounts (9).

***S. paradoxus* normalization factor**

Number of mapped read segments were calculated using SAMtools idxstats (10), from which number of reads assigned to the *S. paradoxus* genome were recovered. Total number of *S. paradoxus* reads for each sample was collected, excluding the rDNA due to its vast variability in copy number.

**Peak filtering for anchor away experiment**

We evaluated goodness of fit by calculating the standard-deviation of the residuals for each peak (figure S4A). Those peaks that did not fit the non-linear regression model were excluded from further analysis (figure S4B,C). We found 227 Rap1 peaks that were centered over tRNA genes or Ty elements (figure S4A). These loci all displayed relatively short apparent residence times and fit the non-linear regression model well (figure S4A,B). Despite this, we excluded them from further analysis as there is no known connection between Rap1 and Pol III transcribed genes, and it is known that highly transcribed loci are often artifacts of hyper-ChIPability (11, 12). The 377 peaks used in the analyses for figures 5, S3, and S4 represent peaks that fit the non-linear regression model and were within 300bp upstream of an ORF and/or located in the subtelomeric region (defined as 15kb from the ends of chromosomes).

**TIF-seq and NET-seq datasets (previously published)**

To visualize summary distribution plots of elongating RNA polymerase II signal as they related to Rap1 binding sites and dwell-time, we first defined full transcripts spanning from transcription start sites (TSSs) to poly-A tracts by identifying the most abundant, stable transcript isoforms in a dataset generated by TIF-seq (Transcript IsoForm sequencing; GEO Accession GSE39128) (13). We then averaged the corresponding Native Elongating Transcript sequencing signal (NET-seq) from four biological replicates in GEO Accession GSE159603 (14). The positions of the full transcripts were also used to define the regions shown in Figure S2.

Figure S1

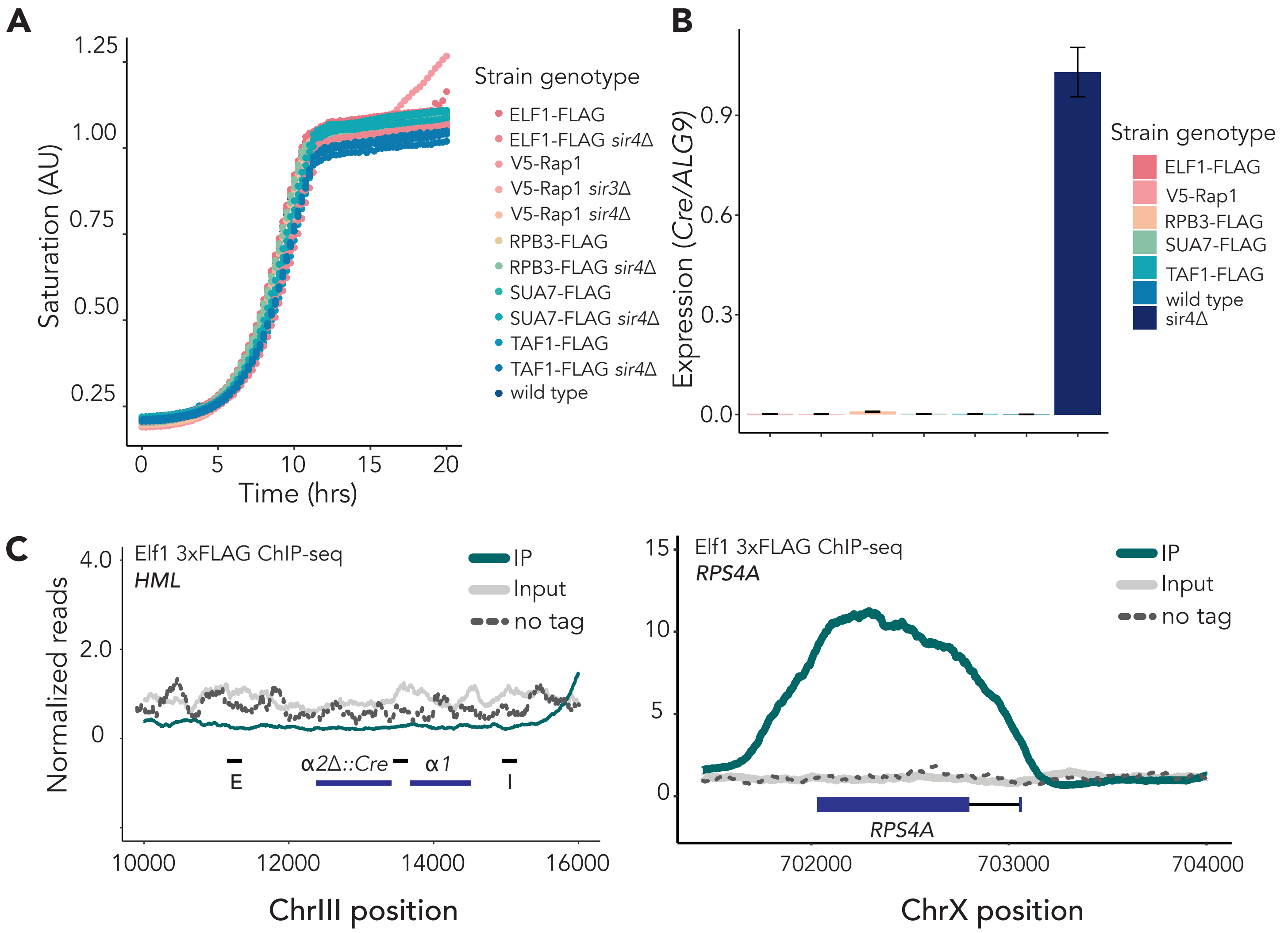


**Figure S1.** Validation of endogenously-tagged strains showed no silencing or growth defects.

A. Growth curves for representative strains for each of the genotypes listed over 20 hours.
B. RT-qPCR quantification of Cre expression at hmlα2Δ::Cre in representative strains for each of the genotypes listed, normalized to the control locus ALG9. Error bars ± SD.
C. Averaged normalized reads for ChIP-seq of two Taf1-3xFLAG samples at HML (left) and RPS4A (right) in SIR cells. Black bars represent 200 bp surrounding Rap1 binding sites at HML-E, HML-p and HML-I, respectively. IP samples are shown in dark green, input values are in light grey, no tag control values are in dark gray. Coverage for only one sample is plotted for input. IP, input, and no tag control are plotted on the same scale.

Figure S2

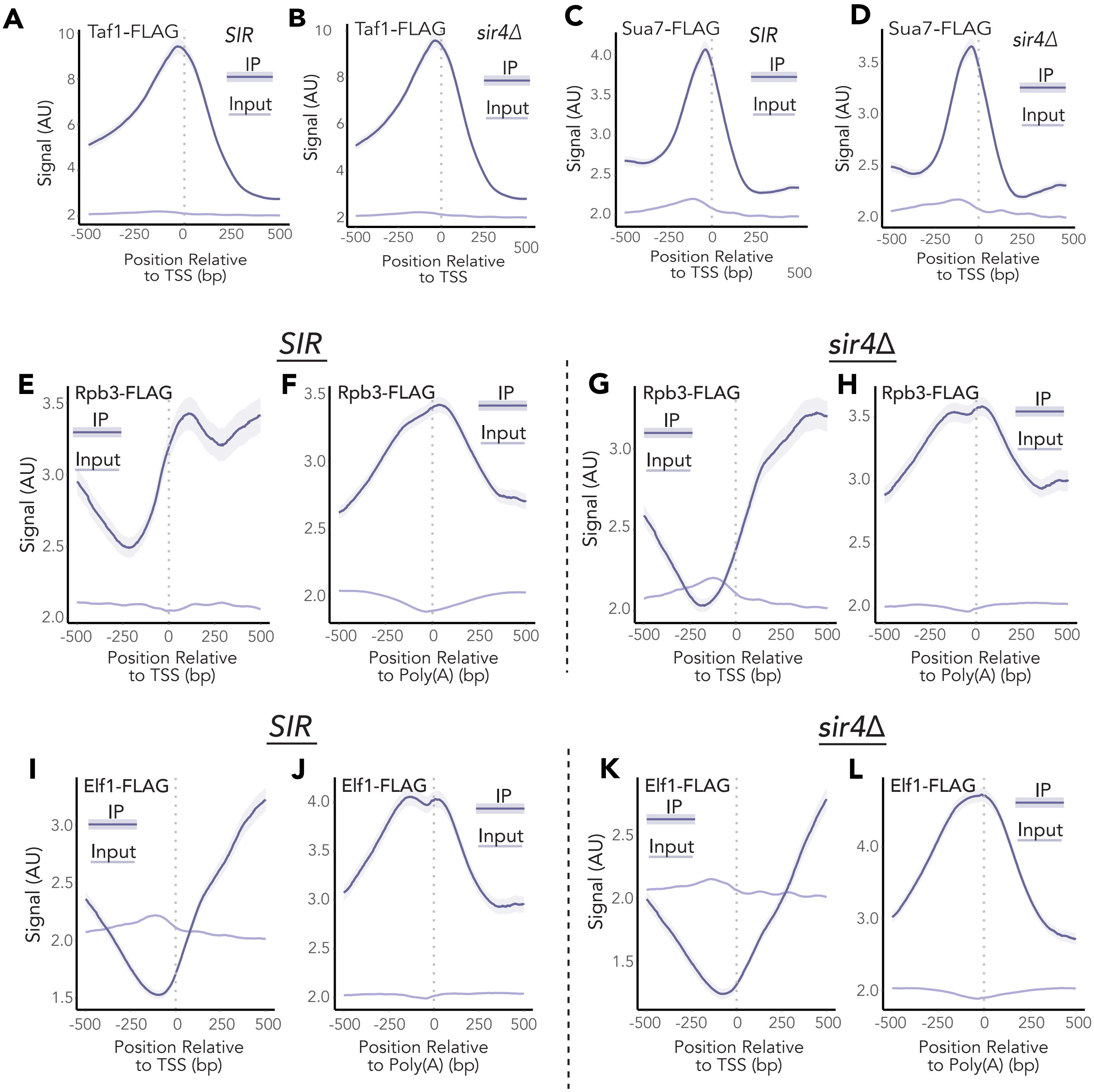
A
Taf1-FLAG
SIR
IP
Input
Signal (AU)
Position Relative to TSS (bp)
B
Taf1-FLAG
sir4Δ
IP
Input
Signal (AU)
Position Relative to TSS
C
Sua7-FLAG
SIR
IP
Input
Signal (AU)
Position Relative to TSS (bp)
D
Sua7-FLAG
sir4Δ
IP
Input
Signal (AU)
Position Relative to TSS (bp)
SIR
sir4Δ
E
Rpb3-FLAG
IP
Input
Signal (AU)
Position Relative to TSS (bp)
F
Rpb3-FLAG
IP
Input
Position Relative to Poly(A) (bp)
G
Rpb3-FLAG
IP
Input
Signal (AU)
Position Relative to TSS (bp)
H
Rpb3-FLAG
IP
Input
Position Relative to Poly(A) (bp)
SIR
sir4Δ
I
Elf1-FLAG
IP
Input
Signal (AU)
Position Relative to TSS (bp)
J
Elf1-FLAG
IP
Input
Position Relative to Poly(A) (bp)
K
Elf1-FLAG
IP
Input
Signal (AU)
Position Relative to TSS (bp)
L
Elf1-FLAG
IP
Input
Position Relative to Poly(A) (bp)

**Figure S2.** Summary distribution plots of each FLAG-tagged transcription associated protein show expected enrichment over promoters and gene bodies.

In each plot, IP signal is plotted in dark purple, paired DNA input signal is plotted in light purple on the same scale. TSS and Poly(A) were defined from (13) as described in the Supplementary Methods above. Shaded regions around the IP values represent 95% CI.

A. The signal average of two Taf1-FLAG ChIP-seq experiments plotted -500 to +500 bp around the transcription start site (TSS) in *SIR* cells.
B. Same as (A) in *sir4*Δ cells.
C. The signal average of two Sua7-FLAG ChIP-seq experiments plotted -500 to +500 bp around the transcription start site (TSS) in *SIR* cells.
D. Same as (C) but in *sir4*Δ cells.
E. The signal average of two Rpb3-FLAG ChIP-seq experiments plotted -500 to +500 bp around the transcription start site (TSS) in *SIR* cells.
F. The signal average of two Rpb3-FLAG ChIP-seq experiments plotted -500 to +500 bp around the Poly(A) tract in *SIR* cells.
G. Same as (E) but in *sir4*Δ cells.
H. Same as (F) but in *sir4*Δ cells.
I. The signal average of two Elf1-FLAG ChIP-seq experiments plotted -500 to +500 bp around the transcription start site (TSS) in *SIR* cells.
J. The signal average of two Elf1-FLAG ChIP-seq experiments plotted -500 to +500 bp around the Poly(A) tract in *SIR* cells.
K. Same as (I) but in *sir4*Δ cells.
L. Same as (J) but in *sir4*Δ cells.

Figure S3

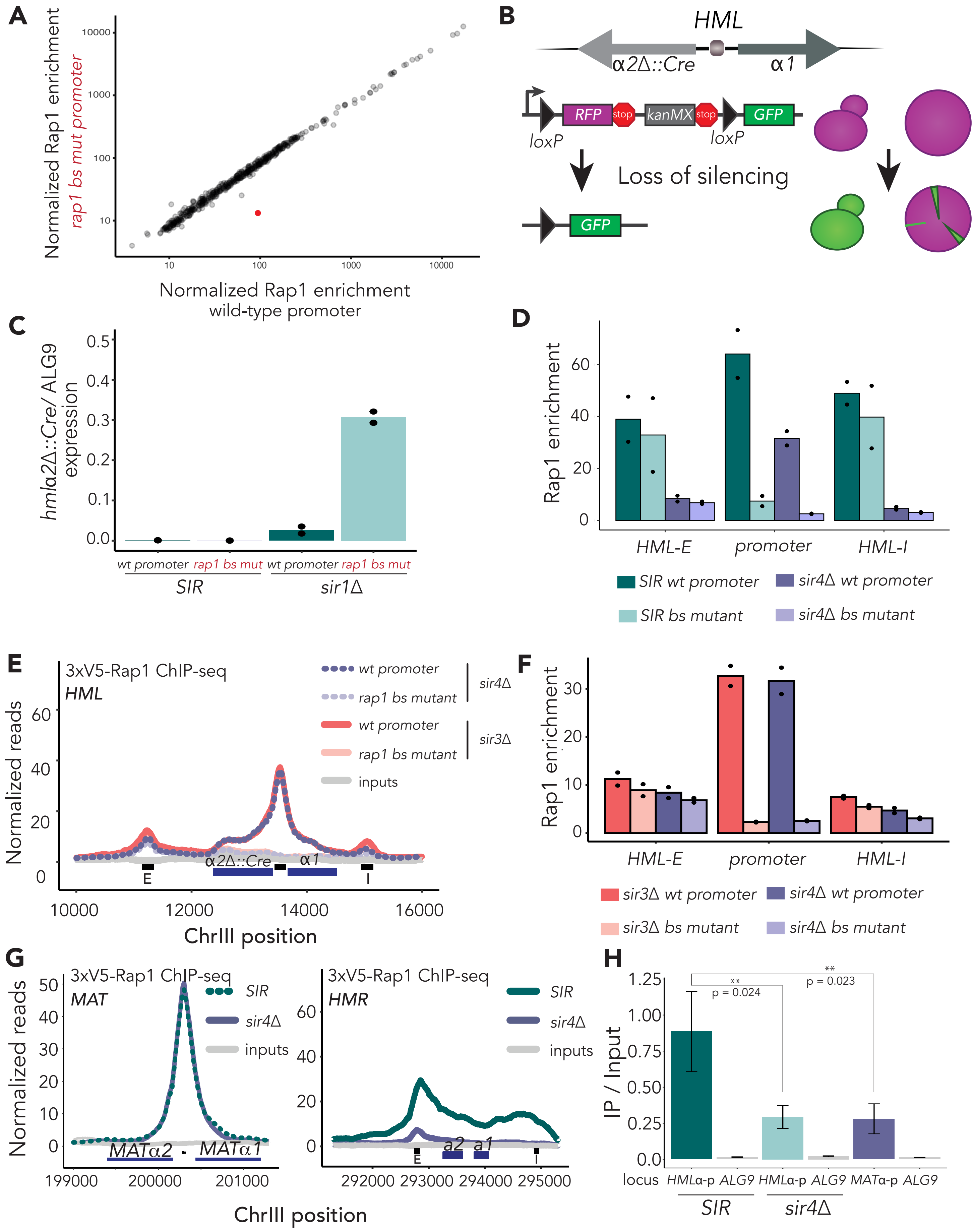
A
Normalized Rap1 enrichment
rap1 bs mut promoter
Normalized Rap1 enrichment
wild-type promoter
B
HML
α2Δ::Cre
α1
RFP
stop
kanMX
stop
GFP
loxP
loxP
Loss of silencing
GFP
C
hmlα2Δ::Cre/ ALG9 expression
wt promoter
rap1 bs mut
wt promoter
rap1 bs mut
SIR
sir1Δ
D
Rap1 enrichment
HML-E
promoter
HML-I
SIR wt promoter
sir4Δ wt promoter
SIR bs mutant
sir4Δ bs mutant
E
3xV5-Rap1 ChIP-seq
HML
Normalized reads
wt promoter
rap1 bs mutant
sir4Δ
wt promoter
rap1 bs mutant
sir3Δ
inputs
α2Δ::Cre
α1
E
I
ChrIII position
F
Rap1 enrichment
HML-E
promoter
HML-I
sir3Δ wt promoter
sir4Δ wt promoter
sir3Δ bs mutant
sir4Δ bs mutant
G
3xV5-Rap1 ChIP-seq
MAT
SIR
sir4Δ
inputs
MATα2
MATα1
3xV5-Rap1 ChIP-seq
HMR
SIR
sir4Δ
inputs
E
a2
a1
I
Normalized reads
ChrIII position
H
IP / Input
**
p = 0.024
**
p = 0.023
locus
HMLα-p
ALG9
HMLα-p
ALG9
MATα-p
ALG9
SIR
sir4Δ

**Figure S3**. Rap1 bs mutant uniquely affects binding and silencing at *HML-p*

A. Normalized Rap1 ChIP signal for top 500 peaks as defined by MACS in cells with or without the rap1 bs mutation at *HMLp*. Plotted values represent the average of two biological replicates. The peak corresponding to *HMLp* is shaded in red and is the most significantly different between the two.
B. CRASH assay experimental design. In this strain, *HMLα2* is replaced with Cre recombinase (*hmlα2*∆*::*Cre). A cassette in which an *RFP* and a selectable marker are flanked by loxP sites and driven by the strong *TDH3* promoter resides at the unrelated *URA3* locus. Downstream of loxP-RFP-kanMX-loxP is GFP with no promoter. Upon loss of silencing at *HML*, *Cre* expression induces an irreversible switch from RFP-kanMX expression to GFP expression (supp fig 2B). In a colony, GFP-positive sectors represent a loss-of-silencing event that was enough to allow expression of even one Cre transcript in the cell at the vertex of the sector, with the subsequent progeny represented in the growth outwards.
C. RT-qPCR of *Cre* transcripts from *hmlα2*∆*::Cre* in *SIR* and *sir1*∆ cells, with and without the rap1 bs mutation at the promoter. mRNA quantification was normalized to *ALG9* expression. Black dots represent individual replicates with the mean shown as colored bars.
D. Quantification of Rap1 enrichment from figure 2A and 2E over each of the three black bars representing *HML-E, HML-I,* and *HML-P.* Black dots represent individual replicates with the mean shown as colored bars.
E. Normalized reads mapped to *HML* in 3xV5-Rap1 ChIP-seq experiments. *sir4*∆ wild-type *HMLp* and rap1 bs mutant *HMLp* cells are in dark and light purple, respectively. *sir3*∆ wild-type *HMLp* and rap1 bs mutant *HMLp* cells are in dark and light pink, respectively. Light grey represents input samples. Each plot is the average of two biological replicates. Light grey represents input samples. Black bars along x-axis represent 200 bp surrounding Rap1 binding sites at *HML-E, HML-p,* and *HML-I,* respectively. IP and input values are plotted on the same scale.
F. Quantification of Rap1 enrichment from (E) over each of the three black bars representing *HML-E, HML-I,* and *HML-P.* Black dots represent individual replicates with the mean shown as colored bars.
G. (Left) Normalized reads mapped to *MAT* in two 3xV5-Rap1 ChIP-seq experiments, averaged. (Right) Normalized reads mapped to *HMR* in two 3xV5-Rap1 ChIP-seq experiments, averaged. Dark green lines represent *SIR* cells. Dark purple lines represent *sir4*∆ cells. Grey lines represent input samples. The Rap1 binding site at the bidirectional promoter is represented by a black line on the x-axis (left). Black bars along x-axis represent 200 bp surrounding *HMR-E, HMR-p,* and *HMR-I,* respectively IP and input values are plotted on the same scale.
H. ChIP-qPCR of 3xV5-Rap1 IP / Input enrichment at the *HML*α-promoter in *SIR* and *sir4*∆*,* and at the *MAT*α promoter, and each at a negative control locus *ALG9.* N = 3; Unpaired t-test p = 0.024 between *HML*α-promoter in *SIR* and *sir4*∆*;* p = 0.023 between *HML*α-promoter and *MAT*α promoter.

Figure S4

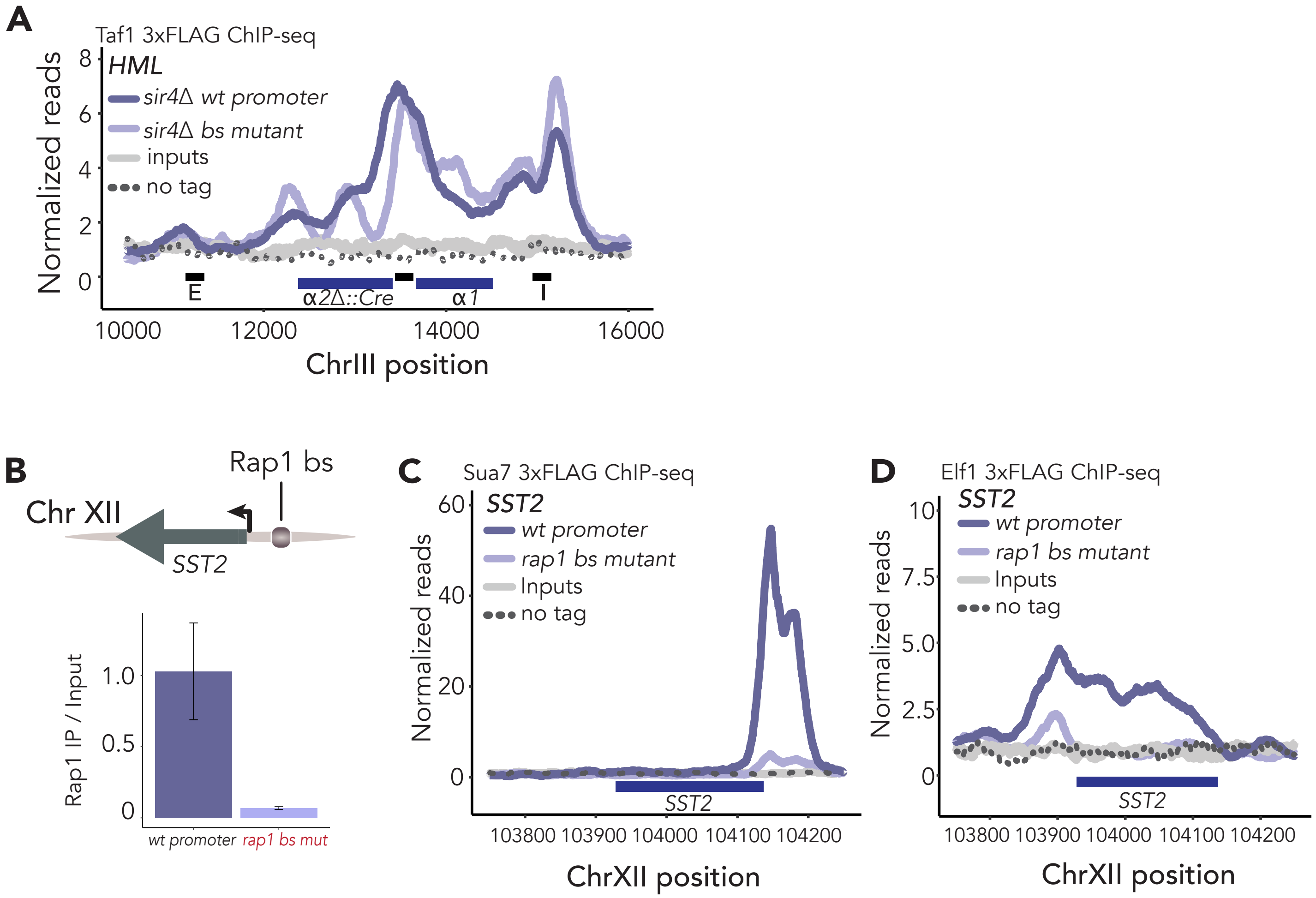


**Figure S4**. The role for Rap1 in promoting transcriptional elongation may be specific to the unique promoter architecture of *HML*.

A. Normalized reads mapped to *HML* in two Taf1-FLAG ChIP-seq experiments for wild-type and mutant Rap1 binding motif at the promoter in sir4Δ cells. IP, input, and untagged controls are plotted on the same scale.
B. (Top) Schematic of the *SST2* locus on ChrXII with a Rap1 binding site indicated. (Bottom) Bar graph representing the data from two V5-Rap1 ChIP-qPCR samples probing the enrichment of Rap1 at a wild type and *rap1 bs mutant* promoter. Rap1 IP is normalized to input DNA quantified at the same locus.
C. Normalized reads mapped to *SST2* in two Sua7-FLAG ChIP-seq experiments for wild-type and mutant Rap1 binding motif at the promoter in *sir4Δ* cells. IP, input, and untagged controls are plotted on the same scale.
D. Same as (C) but for Elf1-FLAG ChIP samples.

Figure S5

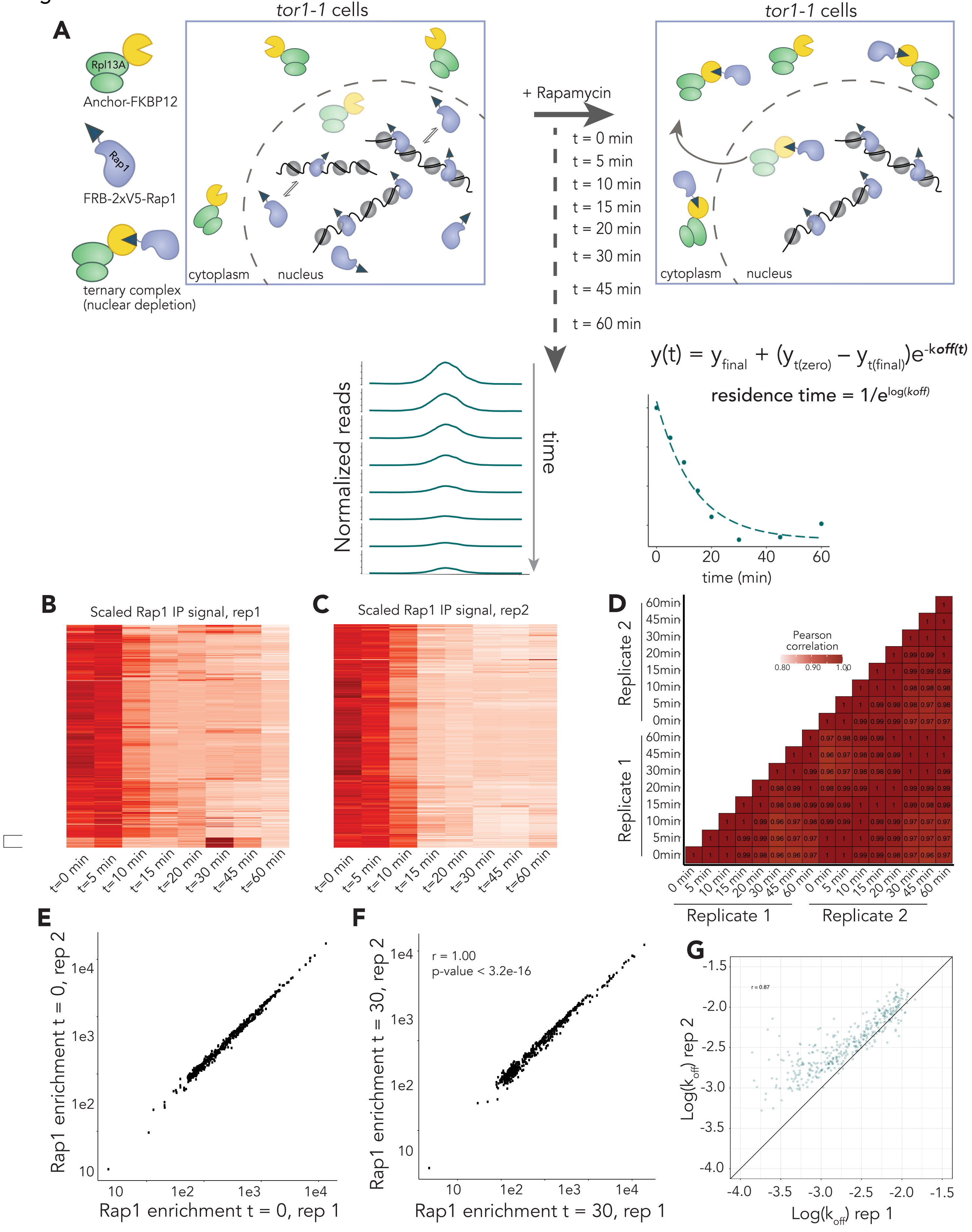

A
tor1-1 cells
Rpl13A
Anchor-FKBP12
Rap1
FRB-2xV5-Rap1
ternary complex
(nuclear depletion)
cytoplasm
nucleus
+ Rapamycin
t = 0 min
t = 5 min
t = 10 min
t = 15 min
t = 20 min
t = 30 min
t = 45 min
t = 60 min
tor1-1 cells
cytoplasm
nucleus
Normalized reads
time
y(t) = y_final + (y_t(zero) − y_t(final))e^-koff(t)
residence time = 1/e^log(koff)
0
20
40
60
time (min)
B
Scaled Rap1 IP signal, rep1
t=0 min
t=5 min
t=10 min
t=15 min
t=20 min
t=30 min
t=45 min
t=60 min
C
Scaled Rap1 IP signal, rep2
t=0 min
t=5 min
t=10 min
t=15 min
t=20 min
t=30 min
t=45 min
t=60 min
D
Pearson
correlation
0.80
0.90
1.00
Replicate 2
Replicate 1
60min
45min
30min
20min
15min
10min
5min
0min
Replicate 1
Replicate 2
E
Rap1 enrichment t = 0, rep 2
Rap1 enrichment t = 0, rep 1
F
r = 1.00
p-value < 3.2e-16
Rap1 enrichment t = 30, rep 2
Rap1 enrichment t = 30, rep 1
G
r = 0.87
Log(k_off) rep 2
Log(k_off) rep 1

**Figure S5.** Design and validation of Rap1 anchor away experiment in biological duplicates.

A. Experimental setup for Anchor Away. A strain harboring a *2xV5-FRB* (FK506 binding protein–rapamycin binding domain) tag at amino-acid 134 in the N-terminus of the protein and the requisite *RPL13a-FKBP12* fusion protein, to which rapamycin binds and establishes an interaction surface for the FRB domain, was constructed. Normalized peaks were fit to a non-linear regression model and the k-off rate was extracted.
B. Clustered heatmaps of normalized peak coverage over the Anchor Away time-course for biological replicate 1.
C. Same as (B) but for biological replicate 2.
D. Pearson correlation coefficients, scaled by color, for each pairwise comparison of each time point in two biological replicates.
E. Correlation between Rap1 enrichment at time = 0 (DMSO) in replicate 1 on x-axis, and Rap1 enrichment at time = 0 in replicate 2 on y-axis. Pearson correlation $r$ = 1.00, p-value < 2.2e-16.
F. Same as (E) but for timepoint t = 30 minutes after addition of rapamycin. Pearson correlation $r$ = 1.00, p-value < 2.2e-16.
G. Correlation between the calculated $\log(k_{off})$) values for all 377 analyzed peaks for biological replicate 1 on x-axis and replicate 2 on y-axis. Pearson correlation $r$ = 0.87.

Figure S6

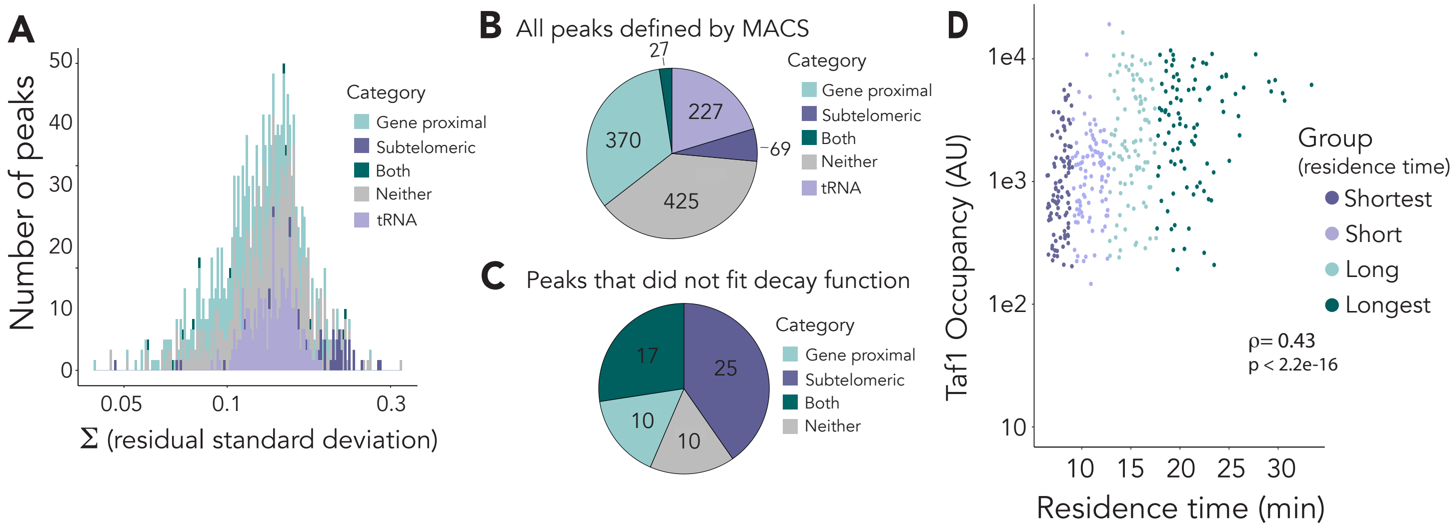


**Figure S6**. Peak filtering and analysis of subtelomeric Rap1 apparent residence times. All data represent the average of two biological replicates.

A. A histogram quantifying the standard-deviation of the residuals for each peak that had a calculated p-value of the log(koff)) < 0.05 (n=1056). Peak classifications are denoted by colors in legend.
B. A pie chart representing the breakdown of peak classifications of all peaks defined by MACS (see Methods) by number (total = 1118).
C. A pie chart representing the breakdown of peaks, by classification, from (C) that did not fit the decay function (p-value of the log(koff)) > 0.05) (total = 62).
D. Correlation between Rap1 apparent residence time (x-axis) and Taf1 enrichment at corresponding Rap1 peaks. Spearman correlation coefficient ρ = 0.43, p-value < 2.2e-16.

**Dataset S1:** Strain table including genotypes for samples used in this study.
**Dataset S2:** Oligonucleotides used in this study including those for strain construction and qPCR.
**Dataset S3:** Chromosomal positions, coverage, and fold difference of Rap1 peaks used for coverage quantifications in figure S2A.
**Dataset S4:** *S. paradoxus* read counts for each sample in the anchor away experiments.
**Dataset S5:** A table containing positional information for 1118 Rap1 peaks, and the normalized count data associated with each.
**Dataset S6:** A table containing positional information, associated genes, apparent residence times, and other characterizations for each of the 377 Rap1 peaks that were analyzed in figure 5.

**SI References**


1. M. E. Gelbart, T. Rechsteiner, T. J. Richmond, T. Tsukiyama, Interactions of Isw2 Chromatin Remodeling Complex with Nucleosomal Arrays: Analyses Using Recombinant Yeast Histones and Immobilized Templates. *Mol Cell Biol* **21**, 2098–2106 (2001).

2. M. Brothers, J. Rine, Mutations in the PCNA DNA Polymerase Clamp of Saccharomyces cerevisiae Reveal Complexities of the Cell Cycle and Ploidy on Heterochromatin Assembly. *Genetics* **213**, 449–463 (2019).

3. D. Goodnight, J. Rine, S-phase-independent silencing establishment in Saccharomyces cerevisiae. *eLife* **9**, e58910 (2020).

4. S. Kubik, *et al.*, Nucleosome Stability Distinguishes Two Different Promoter Types at All Protein-Coding Genes in Yeast. *Molecular Cell* **60**, 422–434 (2015).

5. H. Haruki, J. Nishikawa, U. K. Laemmli, The Anchor-Away Technique: Rapid, Conditional Establishment of Yeast Mutant Phenotypes. *Molecular Cell* **31**, 925–932 (2008).

6. M. Fouet, J. Rine, Limits to transcriptional silencing in Saccharomyces cerevisiae. *Genetics* **223**, iyac180 (2023).

7. G. Monaco, *et al.*, flowAI: automatic and interactive anomaly discerning tools for flow cytometry data. *Bioinformatics* **32**, 2473–2480 (2016).

8. R. Janke, G. A. King, M. Kupiec, J. Rine, Pivotal roles of PCNA loading and unloading in heterochromatin function. *Proc Natl Acad Sci U S A* **115**, E2030–E2039 (2018).

9. Y. Liao, G. K. Smyth, W. Shi, featureCounts: an efficient general purpose program for assigning sequence reads to genomic features. *Bioinformatics* **30**, 923–930 (2014).

10. H. Li, *et al.*, The Sequence Alignment/Map format and SAMtools. *Bioinformatics* **25**, 2078–2079 (2009).

11. P. J. Park, ChIP-Seq: advantages and challenges of a maturing technology. *Nat Rev Genet* **10**, 669–680 (2009).

12. L. Teytelman, D. M. Thurtle, J. Rine, A. van Oudenaarden, Highly expressed loci are vulnerable to misleading ChIP localization of multiple unrelated proteins. *Proc Natl Acad Sci U S A* **110**, 18602–18607 (2013).

13. V. Pelechano, W. Wei, L. M. Steinmetz, Extensive transcriptional heterogeneity revealed by isoform profiling. *Nature* **497**, 127–131 (2013).

14. M. Couvillion, *et al.*, Transcription elongation is finely tuned by dozens of regulatory factors. *eLife* (2022) https:/doi.org/10.7554/eLife.78944 (February 21, 2023).
